# Evaluating the Causal Association Between Type 2 Diabetes and Alzheimer’s Disease: A Two-Sample Mendelian Randomization Study

**DOI:** 10.3390/biomedicines13051095

**Published:** 2025-04-30

**Authors:** Si Han, Tom Lelieveldt, Miriam Sturkenboom, Geert Jan Biessels, Fariba Ahmadizar

**Affiliations:** 1 Department of Data Science and Biostatistics, Julius Global Health, University Medical Center Utrecht, 3584 CX Utrecht, The Netherlands; s.han@umcutrecht.nl (S.H.); m.c.j.sturkenboom@umcutrecht.nl (M.S.); 2 Department of Biomedical Science, University College Utrecht, Utrecht University, 3508 TC Utrecht, The Netherlands; t.s.lelieveldt@students.uu.nl; 3 Department of Neurology, Brain Center, University Medical Center Utrecht, 3584 CX Utrecht, The Netherlands; g.j.biessels@umcutrecht.nl; 4 Department of Epidemiology, Harvard T.H. Chan School of Public Health, Boston, MA 02115, USA

**Keywords:** type 2 diabetes, Alzheimer’s disease, Mendelian randomization

## Abstract

**Background/Objectives**: Type 2 diabetes mellitus (T2DM) and Alzheimer’s disease (AD) are significant global health issues. Epidemiological studies suggest T2DM increases AD risk, though confounding factors and reverse causality complicate this association. This study aims to clarify the causal relationship between T2DM and AD through a systematic review and meta-analysis of Mendelian randomization (MR) studies and a new two-sample MR analysis. **Methods**: A literature search across major databases was conducted through May 2024 to identify MR studies linking T2DM and AD. Fixed/random-effect models provided pooled odds ratios (ORs) with 95% confidence intervals (CIs), and heterogeneity was assessed with the I^2^ statistic. For our MR analysis, we pooled genetic variants from selected studies and analyzed AD outcomes using IGAP, EADB, and UKB databases. Multiple MR methods, including inverse variance weighted (IVW) and pleiotropy–robust approaches, were applied for validation. **Results**: Of 271 articles, 8 MR studies were included (sample sizes: 68,905 to 788,989), all from European ancestry. Our meta-analysis found no significant causal link between T2DM and AD (OR = 1.02, 95% CI: 1.00–1.04) with moderate heterogeneity (I^2^ = 31.3%). Similarly, our MR analysis using 512 SNPs as instrumental variables showed no significant associations in IGAP, EADB, or UKB data, which is consistent across sensitivity analyses. **Conclusions**: This meta-MR and MR analysis revealed no significant causal association between T2DM and AD, indicating that genetic predisposition to T2DM does not appear to causally influence AD risk, though modifiable clinical or environmental aspects of T2DM may still contribute to neurodegenerative processes. Further research should explore other mechanisms linking these conditions.

## 1. Introduction

Type 2 diabetes mellitus (T2DM) is a chronic metabolic condition characterized by impaired insulin sensitivity and persistent hyperglycemia. As a significant public health challenge contributing to an increasing burden worldwide, the global diabetes prevalence in individuals aged 20–79 in 2021 was estimated to be 10.5% (536.6 million people), rising to 12.2% (783.2 million) in 2045 [1]. In addition to its direct health impact and the increasing healthcare costs it brings, T2DM contributes to significant morbidity and mortality through its complications, such as cardiovascular disease, neuropathy, and kidney dysfunction, posing substantial challenges to healthcare systems and public health [1,2,3].

Alzheimer’s disease (AD), the most common form of dementia that contributes to 60–70% of dementia cases [4], is also a prevalent condition that significantly impacts global public health, marked by progressive cognitive decline and neurodegeneration [5].

Numerous epidemiological studies have reported the increased risk of dementia outcomes, especially AD, in individuals with T2DM, suggesting a potential link between these two conditions [6,7]. For instance, a meta-analysis reported a 73% higher risk of all types of dementia and a 56% increased risk of AD in individuals with T2DM [7]. This association has been observed consistently across diverse populations, pointing to T2DM as a significant risk factor for AD. Several biological mechanisms have been proposed to explain this relationship, including insulin resistance, chronic hyperglycemia, and inflammation [8,9,10,11].

Mendelian randomization (MR) analysis is a genetic epidemiology method that infers causal relationships between modifiable risk factors and health outcomes. By leveraging genetic variants as instrumental variables (IVs), MR minimizes confounding and reverse causation, common limitations in observational studies [12]. While MR provides a robust method for inferring causality between T2DM and various complications, there remains a lack of comprehensive synthesis regarding its impact on cognitive outcomes, particularly AD [13,14,15]. Several individual MR analyses have investigated the association between T2DM and AD, but their findings have been inconsistent, and no prior study has systematically synthesized this body of evidence. To address this gap, we conducted a systematic review and meta-analysis of published MR studies on the causal link between T2DM and AD. To complement the meta-analysis and reduce potential bias from duplicated variants, we extracted SNPs associated with T2DM from the included MR studies and removed duplicates. Using this non-overlapping set of SNPs, we conducted an independent two-sample MR to further explore the causal relationship between T2DM and AD.

This integrative framework allowed us to critically evaluate the strength, consistency, and robustness of the causal evidence linking T2DM to AD.

## 2. Materials and Methods

i. **Systematic Review and Meta-MR**

To identify all relevant articles that addressed the causal associations between exposure (T2DM) and outcome (AD), we systematically searched PubMed, Web of Science, and EMBASE regardless of language from inception until 1 May 2024 (the complete search strategy can be found in Supplementary Table S1). The reference list of MR studies included in this review was also searched manually for other potentially relevant inclusions. The Preferred Reporting Items for Systematic Reviews and Meta-Analyses (PRISMA) guidelines were followed. The registered ID in PROSPERO is CRD42024609885.

***Search strategy***

Two authors (SH and TL) independently implemented the search strategy. The process began with an initial screening of titles and abstracts, followed by an in-depth review of the full text for potential articles. Any disagreements between the two reviewers were addressed through discussion with a third author (FA), who provided adjudication.

***Inclusion criteria***

We included MR studies investigating the association between T2DM and AD. Eligible studies were required to report causal estimates, such as odds ratios (ORs) or β-coefficients, presented as an absolute value per unit increase, along with the associated 95% confidence intervals (CIs) or standard errors (SEs). Only full original publications were considered for inclusion. In the case of duplicate cohorts, only the most recent MR studies with unique exposure-genome-wide association studies (GWAS) and outcome-GWAS were retained for meta-MR.

***Data Extraction***

We extracted key details from each eligible MR study, including the first author, publication year, number of IVs, consortiums, sample size, population ancestry, MR design, analysis method, effect metrics (OR with 95% CI or β-coefficients with SEs), and sensitivity MR methods with their results.

***Quality Assessment***

We used the quality assessment tool incorporating ten questions designed to evaluate the quality of MR studies [16]. Among the questions, three are core assumptions of MR: (1) the genetic variants used as IVs must be strongly associated with the exposure of interest, (2) the genetic variants should not be associated with confounding factors, and (3) the genetic variants should influence the outcome solely through their effect on the exposure. Studies failing to address these key assumptions were excluded from the analysis.

***Statistical Analysis***

The effect estimates were combined using either a fixed-effects or random-effects model depending on the heterogeneity among the included studies. Heterogeneity between studies was quantified using the I^2^ statistic with values greater than 75% representing high heterogeneity [17]. For studies reporting β-coefficients and SEs, the ORs and their corresponding CIs were obtained by exponentiating the β-coefficients and their respective CIs.

ii. **Two-sample MR analysis**

We extracted information from eight eligible MR studies included in our systematic review. This included SNPs, major and reference alleles, effect allele frequency, effect size, SEs, effect metrics, *p*-values, closest genes, chromosomes and locations, and sample sizes. One investigator (SH) extracted the data, which were verified by the third investigator (FA). Missing data were requested from corresponding authors via e-mail.

***Instrumental variables selection***

**Figure 1:**
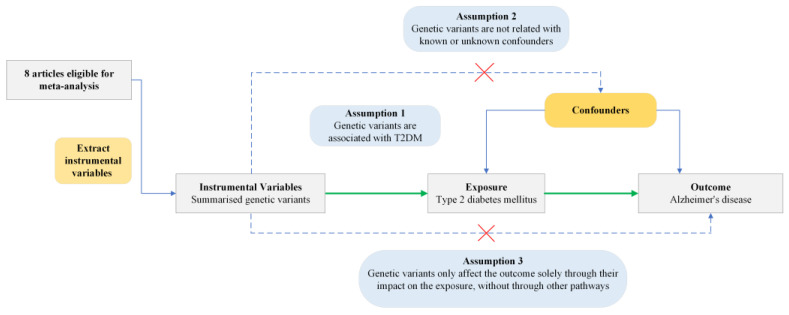
Conceptual framework of Mendelian Randomization analysis assessing the causal effect of type 2 diabetes mellitus on Alzheimer’s disease. T2DM, type 2 diabetes mellitus. Green arrows indicate the assumed causal pathway. Blue arrows show potential confounding paths. Dashed arrows with red crosses represent violations of MR assumptions.

Based on three MR assumptions, we pooled genetic variants demonstrating genome-wide significant associations (*p* < 5 × 10^−8^) with T2DM [16,18]. These variants came from DIAbetes Genetics Replication And Meta-analysis (DIAGRAM), DIAbetes Meta-ANalysis of Trans-Ethnic association studies (DIAMANTE) consortia, and other studies [19,20,21,22,23]. In these GWASs, various definitions of T2DM were used across included studies, commonly based on diagnostic criteria such as fasting glucose (≥7.0 mmol/L), HbA1c (≥6.5%), or non-fasting glucose (≥11.1 mmol/L), or from medical records, hospital discharge data, and electronic health registries. Details of these consortiums can be found in Supplementary Material S1.The study design of the current MR analysis can be found in Figure 1.

Given the overlapping of SNPs across the included studies, we first employed a deduplication strategy based on the *p*-value associated with each SNP’s exposure (T2DM) and the corresponding GWAS sample size. In the final analysis, we retained only non-redundant SNPs, prioritizing those with the lowest *p*-values or derived from the most recent GWAS with the largest cohort size. A total of 1104 SNPs were initially merged from original studies, with 859 proving to be unique. We implemented a standardization process for the effect alleles to ensure comparability and the accurate aggregation of genetic effect estimates across multiple cohorts.

To ensure the validity and robustness of the causal inference and to verify the assumptions underlying the MR approach, associated traits for the SNPs were manually verified using the GWAS catalog and PheWeb. The resulting set of SNPs was then subjected to linkage disequilibrium (LD) clumping using a 1000 kilobases (kb) window and R^2^ < 0.1 (European reference panel). We then calculated F-statistics for each SNP to assess instrument strength, using the formula as follows:

$$F=\frac{\left(N-2\right)\cdot R^{2}}{1-R^{2}}$$

where R was derived from the SNP’s effect size and standard error. SNPs with F ≤ 10 were excluded.

Following this clumping process, 512 SNPs remained. All SNPs exceeded the conventional threshold of F > 10, indicating that the genetic variants explain a significant portion of the variance in the exposure variable. The details of IVs can be found in Supplementary Table S3.

***Outcome Genetic Consortia Data***

The International Genomics of Alzheimer’s Project (IGAP), European Alzheimer & Dementia Biobank (EADB) consortium, and UK Biobank (UKB) from the included MR studies were utilized, which are all publicly available summary-level data [24,25,26]. All the GWAS datasets used in this study obtained relevant ethics committee approvals, and participants informed consent at the time of their original data collection. IGAP is a comprehensive two-stage study based on GWASs of AD in individuals of European descent, which consists of Alzheimer Disease Genetics Consortium (ADGC), European Alzheimer’s Disease Initiative (EADI), and other consortiums [26,27]. In the first stage, IGAP utilized genotyped and imputed data on 7,055,881 SNPs to perform a meta-analysis of four previously published GWAS datasets, which included 17,008 AD cases and 37,154 controls.

EADB united various European cohorts and GWAS consortia, with summary estimates derived from 39,106 participants with clinically diagnosed AD, 46,828 participants with proxy AD, and 401,577 control participants without AD. Proxy AD was determined solely from the UKB through questionnaire data, where participants were asked if they had been diagnosed with AD or dementia.

UKB comprises 500,000 males and females from the general UK population, aged 40–69 at baseline (2006–2010). AD cases were defined as participants identified algorithmically (N = 954), while non-cases comprised those not meeting this criterion (N = 487,331). The analysis was conducted using BOLT-LLM, with adjustments for age, sex, genotyping chip, and the top 10 genetic principal components, in accordance with the Medical Research Council–Integrative Epidemiology Unit UK Biobank GWAS pipeline. Further details about the UK Biobank and the analytic pipeline are available elsewhere [25,28,29].

***Statistical Methods***

To ensure consistency of the effect of allele orientation between exposure and outcome summary statistics, all SNPs were aligned such that effect estimates corresponded to the same reference allele. Strand-incompatible SNPs were removed, and palindromic SNPs (i.e., A/T or C/G variants) were resolved using allele frequency information when available. When allele frequencies were insufficient to determine strand orientation, ambiguous SNPs were excluded to avoid potential misalignment. To enhance instrument coverage in the outcome datasets, proxy SNPs in high LD with the original variants, where the primary SNPs were unavailable in the outcome dataset, were identified and substituted. Variants with a minor allele frequency (MAF) below 0.01 were excluded to reduce the risk of bias associated with low-frequency alleles, and this criterion was applied consistently across all analyses.

In MR analysis, the inverse variance weighted (IVW) method was used as the primary analysis method. The IVW method operates under the assumption that all SNPs included in the causal estimate are valid instruments, implying that they do not violate any of the fundamental assumptions of MR.

To assess the robustness of our MR estimates and detect potential violations of core assumptions, we conducted a series of sensitivity analyses targeting horizonal pleiotropy and heterogeneity.

Cochran’s Q statistic was used to evaluate heterogeneity across SNP-specific causal estimates; excess heterogeneity may indicate the presence of pleiotropy or invalid instruments.

We performed MR-Egger regression, which tests directional pleiotropy through its intercept, where a non-zero intercept suggests that pleiotropic effects are biasing the causal estimate [30,31].

In addition, we evaluated the robustness of our findings by implementing MR-PRESSO (Pleiotropy RESidual Sum and Outlier), which detects and corrects for outlier SNPs with horizontal pleiotropy. This additional analysis helps clarify whether the observed null causal effect is due to a true lack of causality or potential confounding due to shared genetic architecture.

To further test the robustness of our results, we used the weighted median estimator (WME), which allows up to 50% of the SNPs to be invalid instruments, offering a more robust causal estimate when pleiotropy is present [32,33]. The simple mode and weighted mode methods further complement this by assuming that the causal effect is determined by the most frequent estimate among the SNPs, with the weighted mode giving more importance to stronger instruments [34,35].

As an additional sensitivity analysis, we evaluated the robustness of our findings to the choice of LD clumping threshold (R^2^) used during instrument selection. We applied seven R^2^ thresholds ranging from 0.001 to 0.8 to capture different levels of instrument stringency. For each threshold, we selected SNPs based on the corresponding LD pruning criteria, harmonized exposure, and outcome data using three independent AD GWAS datasets and conducted inverse variance weighted MR analyses.

All statistical analyses were performed using the “TwoSampleMR (0.5.10)” package in R Studio (version 2024.04; Posit, PBC, Boston, MA, USA). All *p*-values were two-sided, and *p* < 0.05 was considered suggestive of statistical significance.

## 3. Results

i. **Systematic Review and Meta-MR**

**Figure 2:**
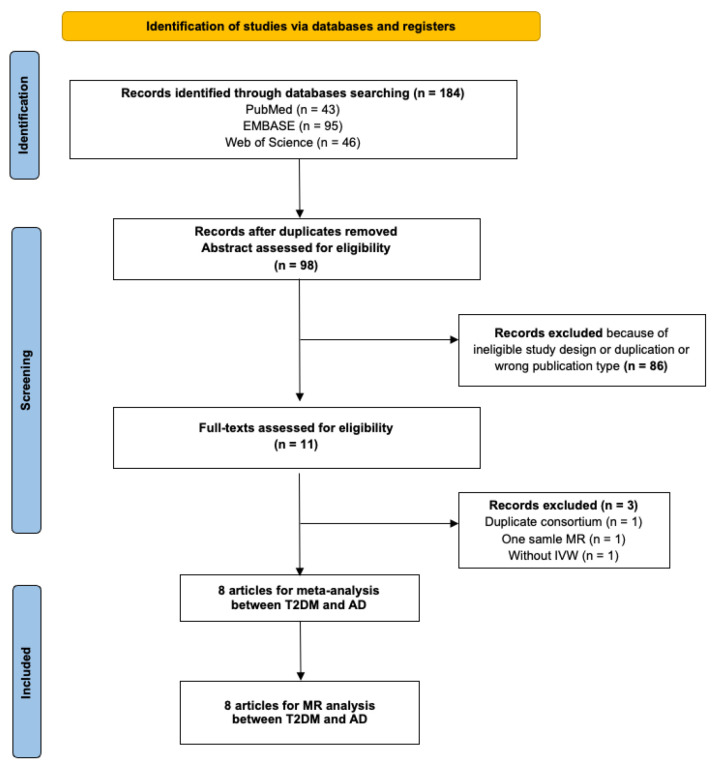
PRISMA (Preferred Reporting Items for Systematic Reviews and Meta-Analyses) flow diagram for systematic review and meta-analysis of association between type 2 diabetes and Alzheimer’s disease. T2DM, type 2 diabetes mellitus; AD, Alzheimer’s disease; MR, mendelian randomization; IVW, inverse variance weighting.

The initial database search yielded 271 articles. Subsequent filtering removed duplicates and articles not meeting the inclusion criteria. Further scrutiny for potential inclusions from reference lists led to 11 articles being considered for the duplication cohort check. Among these, one was excluded because of duplicate exposure and outcome consortium (Morris 2012 GWAS and IGAP); one employed the one-sample MR study design; and one did not use IVW as the primary analysis method. Eight MR studies met all criteria and were selected for inclusion in the meta-MR and subsequent MR analysis [14,36,37,38,39,40,41,42]. In these eight MR studies, total sample sizes, including case and control, range from 68,905 to 788,989, all of European ancestry. All the studies passed the quality assessment. Information on individual studies included in this review (consortium, sample size, IVs, study design, population, and main results) is shown in Supplementary Table S2. The quality assessment questions and results are shown in Supplementary Tables S4 and S5. The PRISMA diagram is shown in Figure 2. The PRISMA diagram is shown in Figure 2. The PRISMA checklist can be found in Supplementary Table S10. The estimates represent the OR of AD per 1-unit higher log odds of T2DM.

**Figure 3:**
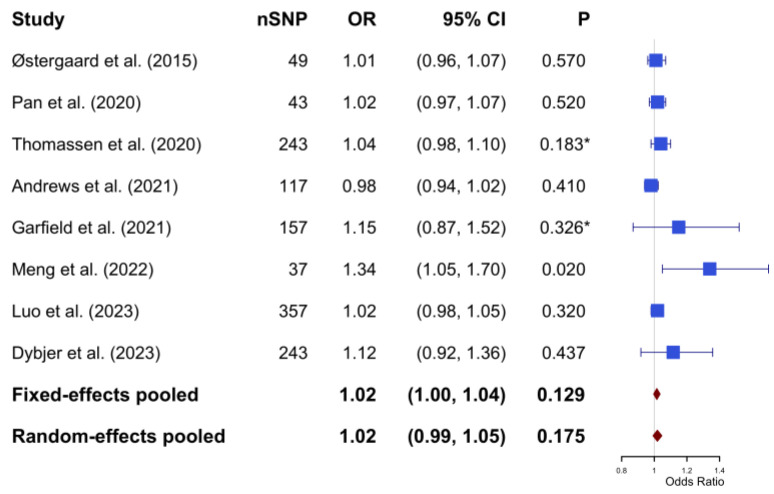
The pooled results of the Mendelian Randomization analysis between type 2 diabetes mellitus and Alzheimer’s disease (based on fixed effect model with heterogeneity I^2^ = 31.3%). * The *p*-values were not reported in the original studies. To enable inclusion in the meta-analysis, *p*-values were calculated based on the reported odds ratio and 95% confidence intervals. The calculation process can be found in Supplementary Material S2. SNP, single nucleotide polymorphism; OR, odds ratio; CI, confidence interval.

In the meta-analysis, Cochran’s Q test yielded a value of 10.74 with a *p*-value of 0.097; the I^2^ statistic was calculated to be 31.3%. Based on a fixed-effect model, the pooled estimate indicated no causal significant association between genetic predisposition to T2DM and the risk of AD (OR: 1.02; 95% CI: 1.00–1.04; *p*-value = 0.129) (Figure 3).

ii. **Two-Sample MR analysis**

Across all three outcome datasets (IGAP, EADB, UKB), we observed no evidence of a significant causal association between T2DM and AD risk using any of the MR methods applied. The inverse variance weighted, weighted median, and MR-Egger methods consistently yielded effect estimates close to the null, with overlapping confidence intervals and non-significant *p*-values. This pattern was consistent across all LD clumping thresholds. The summary of harmonization results across exposure and outcome datasets can be found in Supplementary Table S6. The findings from the MR analyses using each of the three outcome datasets are summarized as follows.

**Figure 4:**
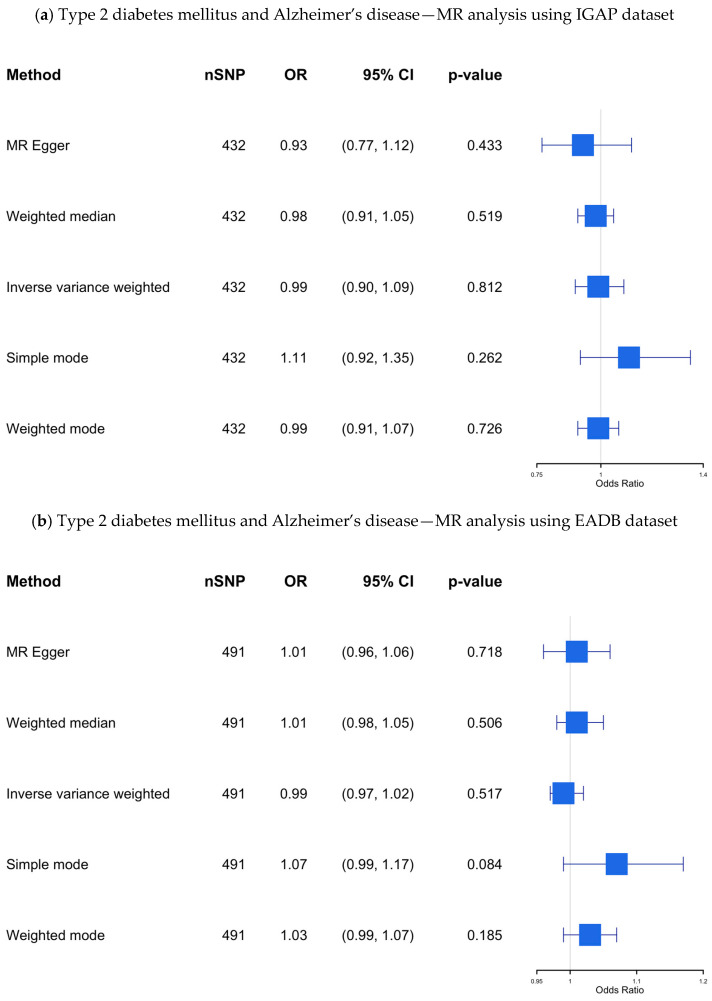

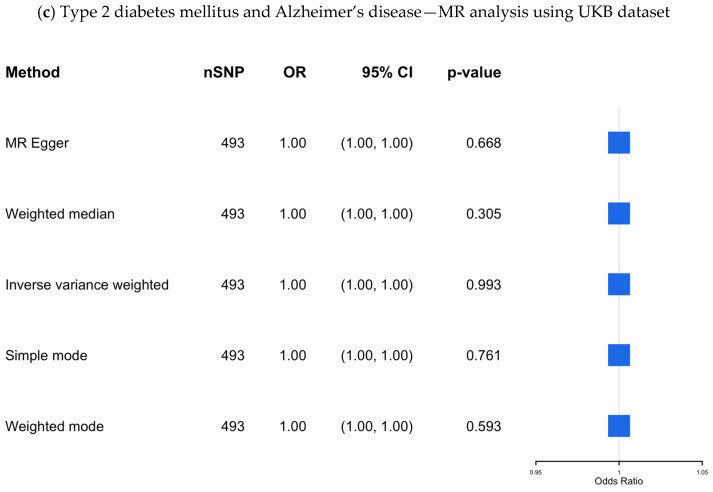
(**a**–**c**) The causal association between type 2 diabetes mellitus and Alzheimer’s disease—MR analysis using the IGAP, EADB, and the UKB datasets. SNP, single nucleotide polymorphism; OR, odds ratio; CI, confidence interval; IGAP, The International Genomics of Alzheimer’s Project; EADB, European Alzheimer & Dementia Biobank consortium; UKB, UK Biobank.

***IGAP dataset:*** The results of our MR study using the IGAP dataset, which included 432 SNPs as IVs, are presented in Supplementary Table S7 and visualized in Figure 4a. Although 512 SNPs were initially identified as IVs after clumping (R^2^ = 0.1), the final analysis included fewer SNPs due to several factors. First, not all SNPs from the clumped list had corresponding outcome data in the outcome dataset, leading to the exclusion of SNPs without matching outcome information. Additionally, despite choosing proxies where possible, some SNPs lacked suitable proxies with sufficient LD, resulting in further reduction.

Our findings revealed no significant causal association (OR 0.989; 95% CI: 0.901–1.085; *p*-value = 0.812) between genetic predisposition to T2DM and AD using the IVW method, even after applying multiple MR methods to assess the robustness of the results.

To evaluate heterogeneity among SNP-specific causal estimates, we calculated Cochran’s Q statistics. Significant heterogeneity was observed for both the IVW (Q = 1059.28, df = 490, *p*-value = 5.63 × 10^−44^) and MR-Egger (Q = 1057.85, df = 489, *p*-value = 5.61 × 10^−44^) models, suggesting potential variability in SNP effects and possible pleiotropy.

To evaluate the presence of horizontal pleiotropy, we applied two complementary approaches. The MR-Egger intercept test, which assesses directional pleiotropy, did not reveal evidence of bias due to unbalanced pleiotropic effects (intercept = 0.0037, SE = 0.0048, *p*-value = 0.44). In contrast, the MR-PRESSO global test detected significant evidence of outlier-driven pleiotropy (residual sum of squares = 3153.365, *p*-value < 0.001). After removing four detected outliers, the outlier-corrected causal estimate remained statistically significant (*β* = 0.0475, SE = 0.0191, *p*-value = 0.013), corresponding to an OR of 1.049 (95% CI: 1.010–1.089). The distortion test did not reveal a significant difference between the original and outlier-corrected estimates (*p*-value = 0.504), suggesting the robustness of the observed association.

**Figure 5:**
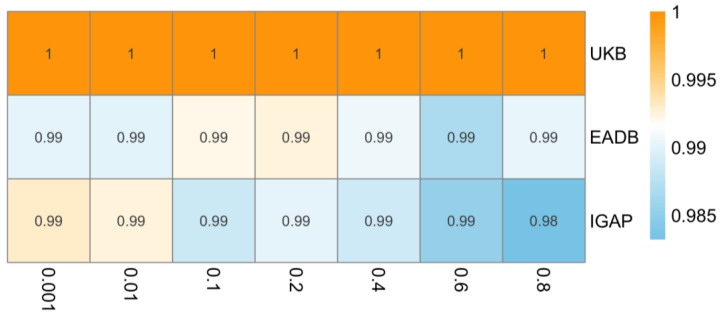
Heatmap of inverse variance weighted odds ratios representing estimated causal effect of type 2 diabetes on Alzheimer’s disease across varying linkage disequilibrium (LD) clumping thresholds (x-axis, R^2^ = 0.001 to 0.8) in different data sources (y-axis). IGAP, The International Genomics of Alzheimer’s Project; EADB, European Alzheimer & Dementia Biobank consortium; UKB, UK Biobank. Note: Odds ratios consistently close to 1 across all LD clumping thresholds suggest no evidence for causal effect of type 2 diabetes on risk of Alzheimer’s disease.

The result remains insignificant in the sensitivity analysis with different clumping R^2^ (ranging from 0.001 to 0.8). As shown in Figure 5, the IVW odds ratios remained broadly consistent across thresholds, without evidence of a significant association.

***EADB dataset:*** The results of this MR study, which included 491 SNPs as IVs, are presented in Supplementary Table S8 and visualized in Figure 4b. No significant causal association was found using the IVW method (OR 0.99; 95% CI 0.97–1.02; *p*-value= 0.52) across all MR methods.

In the Cochran’s Q test, substantial heterogeneity was observed in both IVW (Q = 1059.3, df = 490, *p*-value = 5.63 × 10^−44^) and MR-Egger (Q = 1057.9, df = 489, *p*-value = 5.61 × 10^−44^) analyses, which may indicate the presence of pleiotropic effects or differences in instrument validity.

The MR-Egger intercept suggested no evidence of directional horizontal pleiotropy (intercept = −0.00101, SE = 0.00125, *p*-value = 0.418), indicating that the average pleiotropic effect across SNPs was likely null. We further applied the MR-PRESSO global test, which revealed significant pleiotropy (residual sum of squares = 1075.435, *p*-value < 0.001). A total of 17 SNPs were identified as outliers (e.g., rs10097617, rs1063355, rs11129735), and were removed in a corrected analysis. The outlier-corrected causal estimate remained non-significant (OR = 1.001, 95% CI: 0.98–1.02, *p*-value = 0.89). The distortion test indicated that removing outliers did not significantly change the causal estimate (*p*-value = 0.073), suggesting that horizontal pleiotropy did not substantially bias the MR results.

The result remains insignificant in the sensitivity analysis with different clumping R2 (ranging from 0.001 to 0.8). As shown in Figure 5, the IVW odds ratios showed minimal variation across thresholds, supporting the stability of the null association across different sets of genetic instruments.

***UKB dataset:*** The results from the UKB dataset, which included 493 SNPs as IVs, are presented in Supplementary Table S9 and visualized in Figure 4c, showing no significant association (OR 1.00; 95% CI 1.00–1.00; *p*-value = 0.99).

Cochran’s Q test revealed significant inconsistency in effect sizes for both the IVW (Q = 1319.4, df = 492, *p*-value = 7.96 × 10^−77^) and MR-Egger (Q = 1318.7, df = 491, *p*-value = 5.95 × 10^−77^) models. This suggests that some instruments may be affected by pleiotropic effects or heterogeneity in their associations with the outcome.

In the MR-Egger regression intercept test, the intercept was estimated at 7.89 × 10^−^⁶ (SE = 1.60 × 10^−5^, *p*-value = 0.623), not indicate directional pleiotropy across the included variants.

We also implemented the MR-PRESSO framework, which identified significant pleiotropy (residual sum of squares = 1334.98, *p* < 0.001) based on the global test. Following the exclusion of two SNPs flagged as outliers (rs429358 and rs8071043), the causal effect remained non-significant (OR = 1.00, 95% CI: [1.000–1.000], *p*-value = 0.171). The distortion test comparing estimates before and after outlier removal was non-significant (*p*-value = 0.575), indicating that the detected outliers had minimal influence on the overall MR estimate.

The result remains insignificant in the sensitivity analysis with different clumping R^2^ (ranging from 0.001 to 0.8). As shown in Figure 5, the IVW odds ratios remained identical across all thresholds, indicating high consistency in the null finding regardless of instrument selection.

## 4. Discussion

Our study provides a thorough evaluation of the potential causal association between T2DM and AD by presenting findings of a meta-MR, as well as a new two-sample MR analysis based on IVs for T2DM identified from our review and outcome data from three large datasets (IGAP, EADB, UKB). In the meta-analysis of eight MR studies, we did not observe a statistically significant causal association between genetic predisposition to T2DM and AD. Consistently, our two-sample MR analyses using IVW, MR-Egger, and weighted median methods yielded null results across all three datasets. These findings remained robust across a wide range of instrument selection thresholds (LD clumping R^2^ = 0.001 to 0.8), underscoring the consistency of the null association. The convergence of estimates across multiple methods, datasets, and clumping criteria reinforces the conclusion that there is no evidence for a substantial causal effect of genetic liability to T2DM on AD risk within the limits of our study’s statistical power. Importantly, MR-Egger intercept tests showed no evidence of directional pleiotropy, supporting the validity of our findings. Given that MR leverages genetic variants as proxies for lifelong exposure to risk factors, this method minimizes the influence of confounding and reverse causation, providing stronger causal inference than conventional observational studies. With these results, it is essential to assess the accuracy of our findings and investigate reasons for discrepancies with previous MR studies; one of the included MR studies in our review by Meng et al. [38] reported a significant association between genetically predicted type 2 diabetes and increased risk of Alzheimer’s disease, which contrasts with the null findings of most other studies. Although similar exposure and outcome GWAS datasets were used, methodological differences may account for this inconsistency. Lei Meng et al. selected a relatively small number of SNPs (n = 37) as instrumental variables, which may have introduced weak instrument bias or reduced robustness to pleiotropy. In addition, their analyses were limited to a narrow range of MR methods and lacked extensive sensitivity testing, such as outlier correction or heterogeneity assessment. Furthermore, the study did not explicitly address potential sample overlap or population stratification, which can bias MR estimates if uncorrected. These factors may have contributed to the divergent result. Secondly, the definition of AD can affect the result of the MR analysis. Among the MR studies included in this paper, some studies used proxy AD diagnosis, which may affect the characteristics of the population [14].

While our study did not find strong evidence of a direct causal association, epidemiological studies frequently report links between T2DM and AD. These associations are likely driven by residual confounding factors such as age, obesity, and hypertension, which can contribute to common underlying mechanisms like metabolic dysfunction, inflammation, and vascular damage [6,43]. One possible explanation for the lack of a significant causal link could be that T2DM may influence dementia risk through pathways distinct from those involved in AD, including insulin resistance [44,45,46], chronic hyperglycemia [47,48], inflammation [49,50,51], and vascular dysfunction [52,53,54,55].

T2DM is strongly associated with vascular pathology, including endothelial dysfunction, small vessel disease, and chronic cerebral hypoperfusion, hallmark features of vascular dementia rather than AD [43,56]. These vascular injuries may impair cognitive function through ischemic damage independent of amyloid-β and tau accumulation, which are central to AD pathogenesis [57,58]. Notably, several studies have found that T2DM is more strongly linked to vascular dementia than AD and that cerebrovascular pathology may act via parallel or additive mechanisms rather than as a primary driver of AD [8,56]. While mixed pathologies are common in clinical settings, the divergence in underlying mechanisms supports the plausibility of a null causal association between genetically predicted T2DM and AD risk.

Other studies have found limited evidence of a genetic relationship between T2DM and AD. A large-scale genome-wide cross-trait analysis found no significant genetic correlation between AD and various metabolic traits, including T2DM [59]. Similarly, a recent study reported that a diabetes genetic risk score was not associated with clinically diagnosed AD [60], suggesting limited shared genetic architecture. These findings align with our MR results, reinforcing the conclusion that there is minimal genetic overlap between T2DM and AD. Although we could not directly apply LD Score Regression in our analysis due to data constraints, we acknowledge its value and recommend its use in future research when complete GWAS summary statistics are available.

The coexistence of vascular and neurodegenerative changes in some patients may explain the overlap observed in clinical settings; however, the divergence in underlying mechanisms supports the plausibility of a null finding in the causal association between predicted T2DM and AD risk.

Although our study focused on T2DM as a single exposure, we acknowledge that it is a heterogeneous condition encompassing diverse metabolic dysfunctions. This heterogeneity may obscure distinct causal mechanisms acting through specific pathways. Traits such as insulin resistance, obesity, hyperglycemia, and chronic inflammation are biologically plausible contributors to neurodegeneration and may have differential effects on AD [61,62]. Future MR studies using SNPs associated with specific T2DM-related subphenotypes (e.g., insulin signaling, adiposity, or inflammatory pathways) could help distinguish these effects.

***Clinical Relevance***

The lack of a significant causal association between T2DM and AD in our study suggests that T2DM may not directly contribute to AD development. This finding challenges the common assumption that diabetes is a direct risk factor for AD and necessitates a re-evaluation of the implications of T2DM for cognitive health. However, it is crucial to note that our results do not rule out the possibility that T2DM could increase dementia risk through alternative pathways.

Potential mechanisms, including insulin resistance, chronic hyperglycemia, vascular damage, and inflammation, may independently contribute to cognitive decline, explaining the associations observed in epidemiological studies between T2DM and dementia risk. While our findings do not support a direct link between T2DM and AD, they underscore the need for further research into the broader impact of T2DM on dementia, particularly through non-AD pathways [63,64,65].

***Strengths and Limitations***

A key strength of our study is its comprehensive methodology, which combines a systematic review and meta-analysis of existing MR studies to enhance statistical power and produce robust association estimates. Our original two-sample MR analyses conducted across multiple large-scale datasets further strengthen this.

Another notable strength of our study is the implementation of a comprehensive sensitivity analysis using a range of LD clumping thresholds (R^2^ = 0.001 to 0.8) during instrument selection. This approach allowed us to systematically assess the robustness of our causal estimates under varying assumptions about SNP independence. Stricter thresholds (e.g., R^2^ = 0.001) prioritize including nearly independent variants, minimizing confounding due to LD, and reducing bias, but may lead to fewer instruments and reduced statistical power. In contrast, more permissive thresholds (e.g., R^2^ = 0.8) increase instrument count and power but can introduce correlated variants, potentially biasing causal estimates and inflating standard errors or type I error due to violation of the independence assumption in the LD structure. By demonstrating consistent effect estimates across these thresholds, we provide stronger evidence that our findings are not overly sensitive to the LD structure and are likely to reflect true underlying causal relationships.

However, the study limitations should also be noted. While our IVs were curated from previously published MR studies and filtered through LD clumping and F-statistics (F > 10) to ensure strength and independence, we acknowledge that alternative instrument selection strategies, such as LASSO-based or Bayesian approaches, may enhance robustness, particularly against horizontal pleiotropy. Although these methods were not applied in the current analysis, they represent promising avenues for future methodological refinement. MR estimates reflect lifelong genetic liability to T2DM and may not capture the timing or duration of clinical disease. Since T2DM typically develops in midlife and AD in later life, this temporal mismatch could reduce sensitivity to detect effects active during specific windows. Nonetheless, evidence suggests that midlife T2DM and chronic glycemic dysregulation are key predictors of dementia, and genetic liability is likely to align with the relevant exposure period [11,66,67]. Another consideration is using proxy cases in EADB GWAS, based on parental dementia reports. While increasing statistical power, this may introduce non-differential misclassification and bias results toward the null. To address this, we included two independent outcome datasets (IGAP and UKB) and observed consistent results. Moreover, the EADB study showed similar findings when limited to clinically diagnosed AD cases, further supporting our conclusions [24].

Finally, a limitation of our study is the lack of stratified analyses by subgroups such as sex or age, which may mask potential effect heterogeneity. Future MR studies should explore these dimensions to better understand differential pathways linking T2DM and AD.

## 5. Conclusions

Our comprehensive MR analyses, including both meta-MR and two-sample MR approaches using large and well-powered datasets, found no evidence of a causal association between genetically predicted T2DM and AD. These findings challenge the notion of a direct genetic link between T2DM and AD and underscore the importance of investigating alternative mechanisms, such as shared inflammatory, vascular, or lifestyle-related pathways, that may underlie the observed epidemiological association. Further research is warranted to unravel these complex interconnections and to identify potential targets for prevention and intervention.

## Data Availability

The exposures and outcomes of this study are all from open databases: https://www.ebi.ac.uk/gwas/ (accessed on 1 September 2024).

## Supplementary Materials

The following supporting information can be downloaded at: https://www.mdpi.com/article/10.3390/biomedicines13051095/s1. Supplementary Table S1. Literature search strategy for the systematic review and meta-analysis of Mendelian Randomization studies assessing the association between type 2 diabetes and Alzheimer’s disease; Supplementary Table S2. Characters of the involved papers (among the EUR population); Supplementary Material S1. Information of instrumental variable consortiums; Supplementary Table S3. Information of instrumental variables of type 2 diabetes mellitus; Supplementary Table S4. Quality assessment of each article; Supplementary Table S5. Quality assessment results of eight articles; Supplementary Table S6. Summary of harmonization results across exposure and outcome datasets; Supplementary Material S2. Method for calculating *p*-values from reported odds ratios and confidence intervals; Supplementary Table S7. Result of MR analysis between type 2 diabetes mellitus and Alzheimer’s disease (IGAP dataset); Supplementary Table S8. Result of MR analysis between type 2 diabetes mellitus and Alzheimer’s disease (EDAB dataset); Supplementary Table S9. Result of MR analysis between type 2 diabetes mellitus and Alzheimer’s disease (UKB dataset); Supplementary Table S10. PRISMA (Preferred Reporting Items for Systematic reviews and Meta-Analyses) checklist for systematic review and meta-analysis; Supplementary Table S11. STROBE-MR—Strengthening the Reporting of Observational Studies in Epidemiology using Mendelian Randomization: A checklist of recommended reporting items.
