# Supplementary material for: Evaluating the Causal Association Between Type 2 Diabetes and Alzheimer’s Disease: A Two-Sample Mendelian Randomization Study
Source: Biomedicines. 2025 Apr 30;13(5):1095. doi: 10.3390/biomedicines13051095 (PMC12108868; doi:10.3390/biomedicines13051095)
Supplement: Supplementary file 1 [file biomedicines-13-01095-s001.zip › biomedicines-3564657-supplementary.pdf]

Supplementary Table S1. Literature search strategy for the systematic review and meta-analysis of Mendelian Randomization studies assessing the association between type 2 diabetes and Alzheimer's disease

Supplementary Table S2. Characters of the involved papers (among the EUR population)

Supplementary Material S1. Information of instrumental variables consortiums

Supplementary Table S3. Information of instrumental variables of type 2 diabetes mellitus

Supplementary Table S4. Quality assessment of each article

Supplementary Table S5. Quality assessment results of the 8 articles

Supplementary Table S6. Summary of harmonisation results across exposure and outcome datasets

Supplementary Material S2. Method for calculating p-values from reported odds ratios and confidence intervals

Supplementary Table S7. Result of MR analysis between type 2 diabetes mellitus and Alzheimer's disease (IGAP dataset)

Supplementary Table S8. Result of MR analysis between type 2 diabetes mellitus (T2DM) and Alzheimer's disease (EDAB dataset)

Supplementary Table S9. Result of MR analysis between type 2 diabetes mellitus (T2DM) and Alzheimer's disease (UKB dataset)

Supplementary Table S10. PRISMA (Preferred Reporting Items for Systematic reviews and Meta-Analyses) checklist for systematic review and meta-analysis

Supplementary Table S11. STROBE-MR - Strengthening the Reporting of Observational Studies in Epidemiology using Mendelian Randomization: A checklist of recommended reporting items

Supplementary Table S1. Literature search strategy for the systematic review and meta-analysis of Mendelian Randomization studies assessing the association between type 2 diabetes and Alzheimer's disease

Medline (PubMed), Web of Science, and Embase were searched to identify relevant studies. The keywords applied in the search among the three databases are similar. The search strategy for all databases is presented below.

**Pubmed**	
#1	"diabetes mellitus, type 2"[MeSH Terms] OR "type 2 diabetes"[Title/Abstract] OR "T2DM"[Title/Abstract] OR "T2D"[Title/Abstract] OR "NIDDM"[Title/Abstract] OR "non insulin dependent diabetes mellitus"[Title/Abstract]
#2	"Alzheimer Disease"[MeSH Terms] OR "Alzheimer's disease"[Title/Abstract] OR "Alzheimer"[Title/Abstract] OR "Alzheimer's"[Title/Abstract] OR "AD"[Title/Abstract] OR "Dementia"[MeSH Terms] OR "Dementia"[Title/Abstract]
#3	#1 AND #2
#4	"mendelian randomization analysis"[MeSH Terms] OR "mendelian randomisation"[Title/Abstract] OR "mendelian randomization"[Title/Abstract] OR "genetic instrumental variable"[Title/Abstract] OR "genetic instrument"[Title/Abstract]
#5	(((Editorial[Publication Type]) OR (Letter[Publication Type])) OR (Case Reports[Publication Type])) OR (Comment[Publication Type]) OR (Congress[Publication Type]) OR (Review[Publication Type])
#6	(#3 AND #4) NOT #5

**EMBASE**
1. 'diabetes mellitus, type 2'/exp OR 'type 2 diabetes':ti,ab OR 'T2DM':ti,ab OR 'T2D':ti,ab OR 'NIDDM':ti,ab OR 'non insulin dependent diabetes mellitus':ti,ab
2. 'alzheimer disease'/exp OR 'Alzheimer's disease':ti,ab OR 'Alzheimer':ti,ab OR 'Alzheimer's':ti,ab OR 'AD':ti,ab OR 'dementia'/exp OR 'dementia':ti,ab
3. 1 AND 2
4. 'mendelian randomization analysis'/exp OR 'mendelian randomisation':ti,ab OR 'mendelian randomization':ti,ab OR 'genetic instrumental variable':ti,ab OR 'genetic instrument':ti,ab

5. 3 AND 4<br>6. NOT ([editorial]/lim OR [letter]/lim OR [case report]/lim OR [conference abstract]/lim OR [review]/lim)<br>7. 5 AND 6
**Web of Science**
TS=("diabetes mellitus, type 2" OR "type 2 diabetes" OR T2DM OR T2D OR NIDDM OR "non insulin dependent diabetes mellitus")<br>AND<br>TS=("Alzheimer Disease" OR "Alzheimer's disease" OR "Alzheimer" OR "Alzheimer's" OR AD OR "Dementia")<br>AND<br>TS=("mendelian randomization analysis" OR "mendelian randomisation" OR "mendelian randomization" OR "genetic instrumental variable" OR "genetic instrument")<br>NOT<br>DT=(Editorial OR Letter OR "Case Report" OR Comment OR Congress OR Review)

Supplementary Table S2. Characters of the involved papers (among the EUR population)

	Exposure consortium	Outcome consortium	N	nSNP	MR	Result
Søren D. Østergaard et al, 2015	Morris AP, Voight BF, Teslovich TM, et al. Large-scale association analysis provides insights into the genetic architecture and pathophysiology of type 2 diabetes. Nature genetics. 2012; 44(9):981.	Lambert JC, Ibrahim-Verbaas CA, Harold D, Naj AC, Sims R, Bellenguez C, et al. Meta-analysis of 74,046 individuals identifies 11 new susceptibility loci for Alzheimer's disease. Nat Genet. 2013;45:1452–8.	17,008/ 37,154; 8,572/11,312 (74,046)	49	2SMR	OR [95% CI]: 1.01 [0.96–1.07]; p = 0.57
Yuesong Pan et al, 2020	Morris AP, Voight BF, Teslovich TM, et al. Large-scale association analysis provides insights into the genetic architecture and pathophysiology of type 2 diabetes. Nature genetics. 2012; 44(9):981.<br>Scott RA, Scott LJ, Mägi R, et al. An expanded genome-wide association study of type 2 diabetes in europeans. Diabetes. 2017; 66:2888–902.	Lambert JC, Ibrahim-Verbaas CA, Harold D, Naj AC, Sims R, Bellenguez C, et al. Meta-analysis of 74,046 individuals identifies 11 new susceptibility loci for Alzheimer's disease. Nat Genet. 2013;45:1452–8.	17,008/ 37,154; 8,572/11,312 (74,046)	51	2SMR	OR [95% CI]: 1.02 (0.97-1.07), p = 0.52
Jesper Qvist Thomassen et al, 2020	Scott RA, Scott LJ, Mägi R, et al. An expanded genome-wide association study of type 2 diabetes in europeans. Diabetes. 2017; 66:2888–902.	Lambert JC, Ibrahim-Verbaas CA, Harold D, Naj AC, Sims R, Bellenguez C, et al. Meta-analysis of 74,046 individuals identifies 11 new susceptibility loci for Alzheimer's disease. Nat Genet. 2013;45:1452–8.	17,008/ 37,154; 8,572/11,312 (74,046)	51	2SMR	OR [95% CI]: 1.04 (0.98–1.10)
Victoria Garfield et al, 2021	Mahajan A, Taliun D, Thurner M, et al. Fine-mapping type 2 diabetes loci to single-variant resolution using high-density imputation and islet-specific epigenome maps. Nat Genet. 2018;50(11): 1505-1513.	Bycroft C, Freeman C, Petkova D, et al. The UK Biobank resource with deep phenotyping and genomic data. Nature 2018;562:203–209	488,377	157	2SMR	OR [95% CI]: 1.15 (0.87; 1.52)
Shea J. Andrews et al, 2021	Xue A, Wu Y, Zhu Z, et al. Genome-wide association analyses identify 143 risk variants and putative regulatory mechanisms for type 2 diabetes. Nat Commun 2018;9:2941.	Kunkle BW, Grenier-Boley B, Sims R, et al. Genetic meta-analysis of diagnosed Alzheimer's disease identifies new risk loci and implicates Aβ, tau, immunity and lipid processing. Nat Genet 2019;51:414–430.	35,274/59,163 (94,437)	218	2SMR	Beta (SE) 0.072 (0.017)
Lei Meng et al, 2022	Mahajan A, Wessel J, Willems SM, Zhao W, Robertson NR, Chu AY, et al. Refining the accuracy of validated target identification through coding variant fine-mapping in type 2 diabetes. Nat Genet. 2018;50:559–71.	Lambert JC, Ibrahim-Verbaas CA, Harold D, Naj AC, Sims R, Bellenguez C, et al. Meta-analysis of 74,046 individuals identifies 11 new susceptibility loci for Alzheimer's disease. Nat Genet. 2013;45:1452–8.	17,008/37,154; 8,572/11,312 (74,046)	37	2SMR	OR [95% CI]: 1.34 (1.05, 1.70), p = 0.02
Jiao Luo et al, 2023	Vujkovic M, Keaton JM, Lynch JA, et al.; Discovery of 318 new risk loci for type 2 diabetes and related vascular outcomes among 1.4 million participants in a multi-ancestry meta-analysis. Nat Genet. 2020;52(7):680-691.	Bellenguez C, Küçükali F, Jansen IE, et al.; New insights into the genetic etiology of Alzheimer's disease and related dementias. Nat Genet. 2022;54(4):412-436. doi: 10.1038/s41588-022-01024-z	111,326/677,663 (788,989)	357	2SMR	OR [95% CI]: 1.02 (0.98-1.05)
Elin Dybjer et al, 2023	Mahajan A, Taliun D, Thurner M, et al. Fine-mapping type 2 diabetes loci to single-variant resolution using high-density imputation and islet-specific epigenome maps. Nat Genet. 2018;50(11): 1505-1513.	Manjer J, Carlsson S, Elmståhl S, et al. The Malmö Diet and Cancer Study: Representativity, cancer incidence and mortality in participants and non-participants. Eur J Cancer Prev. 2001;10(6): 489-499.	28,098/40, 807 (68,905)	243	2SMR	Beta=-0.11, se=0.1, p = 0.259

2SMR, Two-sample Mendelian randomization; 1SMR, One-sample Mendelian randomization; IVW, Inverse variance weighting; T2D, type 2 diabetes; OR, odds ratio; CI, confidential interva

Supplementary Material S1. Information of instrumental variables consortiums

DIAbetes Genetics Replication And Meta-analysis (DIAGRAM)

The DIAGRAM stage 1 meta-analyses comprise 26,676 T2DM case and 132,532 control individuals from 18 studies genotyped using commercial genome-wide single nucleotide variant (SNV) arrays. The Metabochip stage 2 follow-up comprises 14,545 T2DM case and 38,994 control subjects (N_{eff} = 38,645) from 16 nonoverlapping stage 1 studies (D2D2007, DANISH, DIAGEN, DILGOM, DRsEXTRA, EMIL-Ulm, FUSION2, NHR, IMPROVE, InterACT-CMC, Leipzig, METSIM, HUNT/TROMSO, SCARFSHEEP, STR, Warren2/58BC). Additional follow-up was conducted in 2,796 T2DM case and 4,601 control subjects from the European Prospective Investigation into Cancer and Nutrition-InterAct (EPIC-InterAct) study and in 9,747 T2DM case and 61,857 control subjects from the Resource for Genetic Epidemiology on Adult Health and Aging (GERA) study (1). 13 novel T2DM-associated loci ($P < 5 \times 10^{-8}$) were identified, including variants near the *GLP2R*, *GIP*, and *HLA-DQA1* genes (1). Various definitions of type 2 diabetes mellitus used across included studies, commonly based on diagnostic criteria such as fasting glucose (≥ 7.0 mmol/L), HbA1c (≥ 6.5%), or non-fasting glucose (≥ 11.1 mmol/L). Diagnostic methods include self-reported physician diagnoses, medical records, hospital discharge data, and electronic health registries. Some definitions also require treatment with diabetes medication. Specific criteria like the exclusion of cases with markers for type 1 diabetes (e.g., antibodies) or the use of American Diabetes Association and WHO standards are also mentioned. Controls were typically individuals with glucose levels below diagnostic thresholds and no diabetes diagnosis or treatment history, often matched to cases by demographics and health metrics (1).

DIAbetes Meta-ANalysis of Trans-Ethnic association studies (DIAMANTE)

The DIAMANTE Consortium was established to assemble T2DM GWAS across diverse ancestry groups. Analyses of the European and East Asian ancestry components of DIAMANTE have previously been reported. In the GWAS included in current study, coding variant data was aggregated for 81,412 T2DM cases and 370,832 controls of diverse ancestry through performing both European-specific (EUR) and trans-ethnic (TE) meta-analyses, identifying 40 coding variant association signals (P < 2.2 × 10-7); of these, 16 map outside known risk-associated loci. Genotypes were assembled from: (a) 58,425 cases and 188,032

controls genotyped with the exome-array; (b) 14,608 cases and 174,322 controls from UK Biobank and GERA (Genetic Epidemiology on Adult Health and Aging) genotyped with GWAS arrays enriched for exome content and/or coverage of low-frequency variation across ethnic groups; and (c) 8,379 cases and 8,478 controls with whole-exome sequence from GoT2DM/T2DM-GENES and SIGMA studies (2-6). T2DM diagnosis criteria across studies generally include prior T2DM diagnosis, fasting glucose levels equal to or exceeding 7 mmol/L, HbA1c levels above 6.5%, or the use of glucose-lowering medications. For prevalent and incident cases, ome studies use criteria from established guidelines such as the American Diabetes Association, WHO, or cohort-specific frameworks (e.g., CHS, CMS). In some settings, the criteria extend to 2-hour glucose tests (values above 11.1 mmol/L) or casual glucose measurements when fasting data is unavailable. Diagnosis sources vary from self-reported cases, physician assessments, medication records, or laboratory-confirmed hyperglycemia, with additional specifications in some cohorts, such as C-peptide levels or the absence of anti-GAD antibodies (2).

Another latest multi-ancestry meta-analysis was also applied in the included MR studies. Type 2 diabetes genetic susceptibility via multi-ancestry meta-analysis of 228,499 cases and 1,178,783 controls in the Million Veteran Program (MVP) and other studies with non-overlapping participants: DIAMANTE Consortium, Penn Medicine Biobank, Pakistan Genomic Resource, Biobank Japan, Malmö Diet and Cancer Study, Medstar, and PennCath was investigated. In this study, 568 associations, including 286 autosomal, 7 X-chromosomal and 25 identified in ancestry-specific analyses that were previously unreported was reported. The diagnosis of type 2 diabetes varies from clinical and administrative criteria, including physician diagnosis, self-reports, to biochemical measures. Core diagnostic criteria involve fasting glucose levels ≥ 7.0 mmol/L, HbA1c ≥ 6.5%, and/or the use of glucose-lowering medications. Some studies rely on specific diagnostic codes (such as ICD-9) or criteria from the WHO or ADA. Advanced protocols include exclusion of Type 1 diabetes through age criteria, antibody testing (e.g., GAD antibodies), and C-peptide levels, while others apply a multi-source approach, verifying diagnosis across electronic health records, registries, or hospital records (7).

Supplementary Table S3. Information of instrumental variables of type 2 diabetes mellitus

SNP	chr	pos	A1	A2	eaf	beta	se	pval	gene
rs12741141	1	6669970	G	C	0.362	0.040	0.005	1.37E-16	KLHL21
rs4845987	1	11306279	C	G	0.692	0.028	0.005	2.78E-08	MTOR
rs10916784	1	20729451	G	C	0.585	0.027	0.005	3.91E-09	LINC01141
rs6685701	1	26868639	A	G	0.279	0.030	0.005	1.14E-08	RPS6KA1
rs111824905	1	28110797	C	T	0.055	0.069	0.012	4.02E-09	STX12
rs10915188	1	29024956	A	G	0.585	0.029	0.005	3.69E-10	GMEB1
rs61779275	1	39820310	T	C	0.220	0.075	0.006	3.35E-43	MACF1
rs3176466	1	51438365	C	T	0.903	0.064	0.008	9.36E-16	CDKN2C
rs2269247	1	64107284	C	T	0.814	0.035	0.006	5.74E-09	PGM1
rs10889560	1	65989878	A	C	0.088	0.047	0.008	4.01E-09	LEPR
rs4655617	1	67010654	C	A	0.434	0.028	0.005	2.45E-09	SGIP1
rs2613503	1	72839774	A	C	0.801	0.039	0.006	7.48E-11	RNU6-1246P,RPL31P12
rs197379	1	112292303	C	T	0.387	0.027	0.005	9.14E-09	INKA2
rs1127215	1	117532790	C	T	0.585	0.043	0.005	1.93E-20	PTGFRN
rs41276588	1	118148384	A	G	0.283	0.038	0.005	4.10E-13	TENT5C-DT
rs1493694	1	120526982	T	C	0.107	0.071	0.007	6.51E-22	NOTCH2
rs72692805	1	149894355	G	A	0.924	0.054	0.009	6.01E-10	SV2A,SF3B4
rs145904381	1	151017991	T	C	0.990	0.174	0.023	4.31E-14	BNIPL
rs1194606	1	154294260	C	T	0.233	0.030	0.006	3.41E-08	AQP10
rs3020781	1	155269776	G	A	0.270	0.033	0.005	1.05E-10	PKLR
rs4916253	1	172361032	G	T	0.431	0.027	0.005	3.09E-09	PIGC,DNM3
rs539515	1	177889025	C	A	0.200	0.051	0.008	1.20E-10	NA
rs567185	1	201763499	T	C	0.634	0.037	0.005	1.55E-14	NAV1,IPO9-AS1
rs12048743	1	205114873	G	C	0.440	0.032	0.005	2.11E-12	DSTYK
rs7538321	1	205789455	T	A	0.125	0.042	0.007	1.33E-09	PM20D1-AS1
rs2336938	1	206618799	A	C	0.484	0.030	0.005	9.54E-11	SRGAP2
rs79687284	1	214150821	C	G	0.035	0.148	0.020	2.60E-16	PROX1
rs3738430	1	214157546	G	A	0.976	0.130	0.021	2.80E-09	NA
rs340874	1	214159256	C	T	0.553	0.067	0.005	5.37E-48	PROX1,PROX1-AS1
rs2820444	1	219741820	G	A	0.713	0.047	0.005	8.66E-21	ZC3H11B
rs348330	1	229672955	G	A	0.369	0.053	0.007	6.50E-16	ABCB10

rs291367	1	235690800	G	A	0.630	0.044	0.007	6.10E-10	NA
rs62107261	2	422144	T	C	0.954	0.113	0.016	3.80E-12	TMEM18
rs10188334	2	653874	C	T	0.828	0.050	0.006	7.76E-16	TMEM18,LINC01875
rs11680058	2	16574669	A	G	0.838	0.056	0.010	1.23E-08	CYRIA,GACAT3
rs7558413	2	18721662	A	G	0.570	0.029	0.005	6.44E-10	KCNS3,RDH14
rs34845373	2	25635771	A	G	0.729	0.037	0.005	1.18E-12	DTNB
rs72803684	2	26192802	T	C	0.046	0.069	0.012	6.01E-09	KIF3C
rs1260326	2	27730940	C	T	0.595	0.064	0.005	2.55E-42	GCKR
rs4952673	2	43423870	A	G	0.470	0.039	0.006	1.60E-09	NA
rs13414140	2	43671176	C	T	0.886	0.118	0.007	4.30E-60	THADA
rs10193538	2	58981064	T	G	0.610	0.039	0.007	8.90E-09	BNIPL
rs980183	2	59311536	G	A	0.394	0.036	0.005	1.02E-14	LINC01122,LINC01793
rs243018	2	60586707	G	C	0.463	0.056	0.005	1.52E-33	MIR4432HG
rs2540949	2	65284231	A	T	0.616	0.050	0.005	9.19E-26	CEP68
rs2028150	2	65655012	C	G	0.598	0.049	0.007	2.30E-12	CEP68
rs1430780	2	67878328	T	C	0.323	0.027	0.005	3.35E-08	LINC02831,LINC01812
rs34506349	2	100598726	G	A	0.960	0.068	0.012	1.76E-08	AFF3
rs17624303	2	105148418	C	T	0.725	0.029	0.005	4.88E-08	LINC01102
rs72836348	2	111888043	G	A	0.892	0.056	0.008	3.20E-13	BCL2L11,MIR4435-2HG
rs34589210	2	112795492	A	G	0.143	0.039	0.007	6.89E-09	TMEM87B,MERTK
rs9784137	2	121325908	G	A	0.849	0.061	0.007	5.25E-21	Y_RNA,LINC01101
rs2033159	2	145261174	C	A	0.234	0.035	0.006	4.90E-10	ZEB2
rs7609422	2	146348037	G	A	0.410	0.030	0.005	2.67E-10	RPL6P5,METAP2P1
rs7559658	2	147920213	C	T	0.190	0.034	0.006	3.17E-09	LINC01911,RNU6-692P
rs13020443	2	152167830	C	T	0.498	0.031	0.005	2.65E-11	NMI,TNFAIP6
rs7568172	2	158335340	G	A	0.940	0.067	0.010	2.64E-12	CYTIP
rs6432613	2	161145612	G	A	0.724	0.039	0.005	6.73E-14	RBMS1
rs13389219	2	165528876	C	T	0.603	0.065	0.005	7.35E-44	COBLL1
rs12992995	2	175197545	C	A	0.732	0.031	0.005	2.46E-09	SP9,LINC01305
rs6715901	2	179650954	G	A	0.512	0.027	0.005	3.06E-09	TTN
rs6741676	2	181618654	A	G	0.668	0.032	0.005	3.64E-11	SCHLAP1
rs12463719	2	203450680	A	G	0.282	0.032	0.005	6.15E-10	BMPR2,MTCO1P17
rs34329895	2	208870017	A	G	0.397	0.028	0.005	3.10E-09	PLEKHM3
rs13005841	2	212302573	A	T	0.713	0.029	0.005	1.89E-08	ERBB4

rs17354348	2	213835977	A	G	0.742	0.029	0.005	2.99E-08	MIR4776-1,IKZF2
rs6736415	2	226851035	C	T	0.180	0.047	0.008	9.30E-09	NA
rs2972145	2	227101309	C	T	0.635	0.090	0.005	7.45E-80	MIR5702,NYAP2
rs77402945	2	227225798	C	T	0.850	0.051	0.009	9.00E-09	NA
rs7561798	2	228973660	G	A	0.485	0.028	0.005	1.16E-09	SPHKAP
rs838735	2	234324192	C	G	0.384	0.029	0.005	3.64E-10	DGKD
rs3872707	3	9514016	A	G	0.140	0.045	0.007	3.46E-11	SETD5
rs9826367	3	12294202	A	G	0.560	0.041	0.006	2.90E-10	NA
rs17036160	3	12329783	C	T	0.880	0.103	0.007	3.04E-47	PPARG
rs4465929	3	15741389	T	C	0.404	0.030	0.005	5.11E-11	ANKRD28,BTD
rs35352848	3	23455582	T	C	0.793	0.062	0.006	7.37E-28	UBE2E2
rs118109589	3	23635623	G	A	0.030	0.120	0.018	7.40E-11	NA
rs10490871	3	35667761	G	A	0.369	0.027	0.005	6.58E-09	ARPP21,RNU6-243P
rs11129735	3	36870230	A	G	0.462	0.026	0.005	1.53E-08	TRANK1
rs11926707	3	46925539	C	T	0.626	0.046	0.008	1.69E-08	PTH1R
rs62262091	3	47693664	T	C	0.089	0.056	0.009	1.14E-10	SMARCC1
rs4688760	3	49980596	T	C	0.674	0.034	0.005	1.10E-11	RBM6
rs2581787	3	53127677	T	G	0.558	0.025	0.005	3.14E-08	RFT1
rs76263492	3	54828827	T	G	0.045	0.091	0.016	6.30E-09	NA
rs2292662	3	63897215	C	T	0.844	0.056	0.007	7.64E-18	SCAANT1,ATXN7
rs4132228	3	64708114	C	T	0.703	0.047	0.005	6.22E-21	ADAMTS9-AS2
rs844215	3	71656045	C	T	0.587	0.026	0.005	2.04E-08	FOXP1-DT,EIF4E3
rs11922794	3	72813582	C	G	0.251	0.030	0.005	1.46E-08	SHQ1
rs13085136	3	72865183	C	T	0.928	0.077	0.012	1.50E-08	SHQ1
rs1437055	3	86831077	A	C	0.613	0.027	0.005	1.19E-08	VGLL3,LINC02070
rs11716527	3	89986280	C	T	0.095	0.049	0.009	6.69E-09	MTCO1P6,U3
rs6438247	3	115084080	C	T	0.155	0.044	0.007	4.05E-11	GAP43,EIF4E2P2
rs11708067	3	123065778	A	G	0.771	0.078	0.006	1.50E-46	ADCY5
rs9873519	3	124921457	T	C	0.534	0.038	0.005	3.57E-16	SLC12A8
rs9828772	3	129333182	C	G	0.899	0.052	0.008	2.70E-11	PLXND1,CARMIL2P1
rs1225052	3	131644937	G	A	0.371	0.027	0.005	1.00E-08	CPNE4
rs9852406	3	135625498	T	C	0.254	0.038	0.005	8.96E-13	SDHBP1,EPHB1
rs6766859	3	138055136	C	T	0.376	0.033	0.005	7.23E-12	MRAS,NME9
rs73872717	3	141134569	C	T	0.953	0.086	0.011	4.78E-15	ZBTB38

rs34573045	3	149196752	G	C	0.420	0.031	0.005	2.27E-11	TM4SF4
rs62271373	3	150066540	A	T	0.056	0.069	0.010	4.44E-11	TSC22D2
rs7619041	3	152095371	T	A	0.487	0.043	0.008	2.76E-08	NA
rs74672008	3	152451616	G	A	0.960	0.080	0.012	1.38E-11	ATP5MGP5,MBNL1
rs56394279	3	160171092	C	T	0.468	0.031	0.005	5.30E-12	TRIM59,B3GAT3P1
rs1449348	3	168225055	C	T	0.859	0.045	0.007	6.64E-12	EGFEM1P
rs9873618	3	170733076	G	A	0.709	0.058	0.005	2.41E-30	SLC2A2
rs686998	3	173119768	G	A	0.539	0.027	0.005	6.11E-09	NLGN1
rs2313211	3	183738626	T	A	0.448	0.029	0.005	4.06E-10	ABCC5,EEF1A1P8
rs10937208	3	184877626	G	A	0.136	0.044	0.007	1.06E-10	EHHADH-AS1,C3orf70
rs9854769	3	185520948	G	A	0.319	0.108	0.005	1.06E-107	IGF2BP2
rs113672528	3	185540817	C	A	0.020	0.140	0.021	1.10E-10	NA
rs3887925	3	186665645	T	C	0.547	0.068	0.007	3.10E-22	ST6GAL1
rs6777684	3	187741842	G	A	0.608	0.057	0.005	3.77E-33	LINC01991,LPP-AS2
rs7619708	3	195810187	T	C	0.758	0.033	0.005	3.46E-10	TFRC,LINC00885
rs1531583	4	744972	T	G	0.045	0.099	0.011	7.06E-19	PCGF3
rs72501964	4	1267203	G	T	0.961	0.083	0.013	2.09E-10	CTBP1-DT
rs56337234	4	1784403	C	T	0.505	0.041	0.005	1.88E-18	FGFR3,TACC3
rs362307	4	3241845	T	C	0.075	0.050	0.009	3.77E-08	HTT
rs10937721	4	6306763	C	G	0.588	0.084	0.005	6.31E-70	WFS1,PPP2R2C
rs2011603	4	18025484	A	G	0.725	0.038	0.005	1.59E-13	LCORL
rs11940813	4	20210953	G	A	0.137	0.037	0.007	2.65E-08	SLIT2,RPL21P46
rs10938398	4	45186139	A	G	0.431	0.043	0.005	3.91E-20	PRDX4P1,THAP12P9
rs62310934	4	48880627	C	G	0.611	0.030	0.005	1.37E-10	OCIAD1,OCIAD2
rs17086692	4	53134293	G	T	0.687	0.047	0.008	2.48E-08	SPATA18
rs114447556	4	53207093	T	C	0.083	0.058	0.009	7.79E-11	RNU6-1252P,SPATA18
rs2055997	4	76535086	G	A	0.701	0.031	0.005	6.53E-10	CDKL2
rs11723275	4	77528821	C	A	0.467	0.027	0.005	4.26E-09	SHROOM3
rs10471048	4	83587562	G	C	0.349	0.034	0.005	2.31E-12	SCD5
rs7660000	4	89751858	C	T	0.713	0.031	0.005	1.29E-09	FAM13A
rs7656001	4	91243865	A	G	0.550	0.026	0.005	1.23E-08	CCSER1
rs6821438	4	95091911	A	G	0.531	0.029	0.005	3.66E-10	SMARCAD1-DT
rs3755879	4	96114385	A	G	0.315	0.033	0.005	5.78E-11	UNC5C
rs7695096	4	103932556	C	T	0.521	0.038	0.005	1.27E-16	SLC9B1

rs17035289	4	106048291	C	T	0.171	0.043	0.006	5.70E-12	TET2,RNU6-351P
rs11098676	4	123833154	C	T	0.788	0.054	0.010	2.03E-08	NUDT6
rs12509379	4	129179458	T	G	0.212	0.031	0.006	2.66E-08	PGRMC2,LARP1B
rs1724557	4	137094048	C	A	0.418	0.025	0.005	4.73E-08	RNU1-89P,TERF1P3
rs12505942	4	140906390	T	C	0.662	0.030	0.005	7.03E-10	MAML3
rs75686861	4	145621328	A	G	0.093	0.047	0.008	4.16E-09	AC098588.2,HHIP
rs6819331	4	153504295	C	T	0.680	0.040	0.005	2.21E-16	RPS3AP18,TMEM154
rs28819812	4	157652753	C	A	0.675	0.038	0.005	3.36E-14	LINC02272,PDGFC
rs72695645	4	185713608	G	A	0.860	0.061	0.007	8.66E-20	ACSL1
rs35901985	4	186580062	A	G	0.824	0.035	0.006	1.69E-08	SORBS2
rs17250977	5	14753745	G	A	0.038	0.113	0.016	2.00E-11	ANKH
rs6885132	5	14768092	C	G	0.900	0.078	0.011	9.50E-13	NA
rs1061813	5	14847331	G	A	0.463	0.043	0.007	3.37E-09	ANKH
rs114136102	5	36084426	C	T	0.040	0.072	0.012	2.05E-09	LMBRD2,UGT3A2
rs13155752	5	44680687	C	A	0.397	0.032	0.005	1.12E-11	LINC02224,RN7SL383P
rs152839	5	50145266	C	T	0.576	0.026	0.005	1.25E-08	PARP8,LINC02106
rs12187734	5	51763665	C	T	0.523	0.029	0.005	2.24E-10	MFSD4BP1,RPS17P11
rs3811978	5	52100489	G	A	0.170	0.053	0.009	4.20E-10	NA
rs4865796	5	53272664	A	G	0.682	0.047	0.005	4.64E-21	ARL15
rs256904	5	55810305	T	A	0.730	0.069	0.005	2.55E-39	C5orf67
rs9687832	5	55861595	A	G	0.198	0.077	0.009	1.70E-20	ANKRD55
rs4976033	5	67714246	G	A	0.411	0.028	0.005	1.93E-09	PIK3R1
rs253412	5	74955841	A	G	0.652	0.046	0.005	2.77E-21	ANKDD1B
rs6878122	5	76427311	G	A	0.311	0.055	0.005	4.60E-29	PDE8B,ZBED3-AS1
rs12519500	5	78436905	C	A	0.654	0.038	0.005	1.76E-15	DMGDH
rs7719891	5	86577352	G	A	0.261	0.040	0.007	8.96E-09	RASA1
rs2410767	5	87705268	C	G	0.779	0.033	0.006	6.79E-09	LINC02060,TMEM161B-DT
rs145510090	5	101273694	A	T	0.053	0.100	0.015	1.10E-12	NA
rs62369303	5	101711308	C	T	0.280	0.047	0.007	3.70E-11	NA
rs17154859	5	102290912	T	G	0.320	0.044	0.007	1.40E-10	NA
rs75432112	5	102586407	A	G	0.048	0.134	0.011	5.34E-36	PPIP5K2,MACIR
rs10077431	5	112927686	C	A	0.785	0.049	0.009	4.76E-08	YTHDC2
rs329122	5	133864599	A	G	0.427	0.026	0.005	1.25E-08	JADE2
rs112667817	5	137823156	C	T	0.879	0.061	0.011	5.76E-09	RPL7P19,ETF1

rs890940	5	158026744	T	C	0.211	0.048	0.006	9.78E-18	EBF1,LINC02227
rs9379084	6	7231843	G	A	0.880	0.075	0.008	3.70E-22	RREB1
rs9505086	6	7232186	C	T	0.380	0.048	0.007	2.70E-13	NA
rs727734	6	15475051	A	T	0.748	0.030	0.005	3.06E-08	JARID2
rs9368112	6	19718157	T	C	0.525	0.026	0.005	2.69E-08	LNC-LBCS
rs11964747	6	20485898	C	T	0.810	0.060	0.008	5.10E-13	NA
rs7756992	6	20679709	G	A	0.271	0.122	0.005	5.52E-128	CDKAL1
rs72832338	6	20775412	A	G	0.081	0.090	0.012	9.90E-15	NA
rs4077404	6	20876613	G	A	0.780	0.061	0.008	5.30E-15	NA
rs3094682	6	31264461	C	A	0.806	0.060	0.006	2.37E-23	HLA-C
rs2246618	6	31478986	T	C	0.307	0.051	0.008	1.20E-09	NA
rs2844492	6	31518169	G	A	0.030	0.140	0.022	1.10E-10	NA
rs1063355	6	32627714	G	T	0.602	0.071	0.008	3.72E-19	HLA-DQB1
rs9275184	6	32654714	C	T	0.111	0.098	0.010	5.59E-24	MTCO3P1,HLA-DQB1
rs9296095	6	33542523	T	C	0.803	0.034	0.006	4.75E-09	GGNBP1,BAK1
rs10305420	6	39016636	C	T	0.616	0.032	0.005	2.69E-11	GLP1R
rs34298980	6	40409243	T	C	0.501	0.038	0.007	4.20E-09	LRFN2
rs4714422	6	41012405	G	A	0.239	0.029	0.005	4.80E-08	OARD1
rs11967262	6	43760327	G	C	0.488	0.037	0.005	2.23E-15	VEGFA,LINC02537
rs10456526	6	43814625	A	G	0.290	0.051	0.007	5.00E-13	NA
rs3798519	6	50788778	C	A	0.180	0.050	0.006	5.49E-17	TFAP2B
rs1819564	6	51505337	A	T	0.027	0.076	0.014	3.85E-08	PKHD1
rs60519666	6	107427166	G	A	0.677	0.035	0.005	3.19E-12	BEND3
rs55812705	6	111738793	T	C	0.749	0.031	0.005	7.45E-09	MFSD4B,REV3L
rs72951506	6	118011723	C	T	0.852	0.042	0.007	9.22E-11	NUS1
rs2008027	6	126052359	G	A	0.504	0.027	0.005	4.28E-09	HEY2-AS1
rs11759026	6	126792095	G	A	0.231	0.065	0.007	6.68E-19	CENPW,MIR588
rs12194820	6	127401978	A	T	0.765	0.046	0.006	9.19E-17	RPS4XP9,RSPO3
rs7739842	6	131954797	G	T	0.194	0.033	0.006	1.98E-08	ENPP3
rs1573090	6	137302159	T	G	0.535	0.045	0.005	3.87E-22	NHEG1,RPL35AP3
rs11155073	6	139837128	T	C	0.421	0.030	0.005	4.43E-11	ATP5PBP6,LINC01625
rs197482	6	143069315	C	T	0.616	0.030	0.005	2.64E-10	HIVEP2,ADGRG6
rs9383649	6	153428102	G	A	0.419	0.033	0.005	1.06E-12	RGS17
rs543159	6	160776017	C	A	0.520	0.032	0.005	1.29E-12	SLC22A3

rs4709746	6	164133001	C	T	0.869	0.058	0.007	1.03E-16	QKI
rs4721089	7	1872921	T	C	0.757	0.034	0.006	7.92E-10	MAD1L1
rs798549	7	2760750	C	A	0.282	0.030	0.005	1.46E-08	AMZ1
rs62450857	7	4683258	A	G	0.133	0.039	0.007	1.85E-08	FOXK1,CYP3A54P
rs13237518	7	12269593	A	C	0.414	0.029	0.005	7.72E-10	TMEM106B
rs1122518	7	13900325	C	T	0.473	0.026	0.005	1.25E-08	RBMX2P4,ETV1
rs17168486	7	14898282	T	C	0.181	0.068	0.007	2.30E-17	DGKB
rs2191349	7	15064309	T	G	0.539	0.066	0.005	6.41E-48	AGMO,GTF3AP5
rs38221	7	15926228	T	C	0.260	0.033	0.005	6.62E-10	CRPPA,RPL36AP26
rs583769	7	18331915	A	G	0.251	0.031	0.005	2.69E-09	HDAC9
rs75693095	7	23440057	C	G	0.021	0.112	0.017	1.42E-11	IGF2BP3
rs62451127	7	27971300	G	A	0.945	0.086	0.014	2.20E-09	NA
rs1513272	7	28200097	C	T	0.509	0.081	0.005	3.97E-71	JAZF1
rs917195	7	30728452	C	T	0.769	0.047	0.006	2.71E-17	CRHR2
rs17439448	7	40816653	T	C	0.121	0.040	0.007	2.98E-08	SUGCT
rs2268576	7	44189023	C	T	0.490	0.039	0.006	1.40E-09	NA
rs2908286	7	44234737	T	C	0.171	0.068	0.006	2.64E-29	GCK
rs12539264	7	48839003	G	A	0.282	0.029	0.005	6.62E-09	GDI2P1,LINC02838
rs2876826	7	50581972	G	A	0.215	0.031	0.006	3.33E-08	DDC
rs2103132	7	69782073	C	G	0.251	0.032	0.005	1.88E-09	AUTS2
rs67755137	7	74108135	A	G	0.190	0.033	0.006	2.24E-08	GTF2I-AS1,GTF2I
rs10240790	7	89880949	G	A	0.704	0.028	0.005	4.69E-08	CFAP69
rs534043	7	100312724	G	A	0.874	0.045	0.007	8.03E-10	POP7,EPO
rs1968204	7	102800137	T	C	0.107	0.057	0.008	1.08E-12	RPL23AP95,DPY19L2P2
rs39328	7	103444978	T	C	0.427	0.028	0.005	3.29E-09	RELN
rs13239186	7	117510621	T	C	0.302	0.054	0.009	2.70E-10	CTTNBP2
rs1562398	7	130457931	G	C	0.417	0.041	0.005	1.99E-18	KLF14,LINC-PINT
rs62492368	7	150537635	A	G	0.315	0.034	0.005	6.38E-12	AOC1
rs6459733	7	156930550	G	C	0.666	0.051	0.005	9.66E-25	UBE3C,MNX1-AS1
rs117173251	8	4186731	T	C	0.033	0.077	0.014	2.48E-08	CSMD1
rs6984305	8	9178268	A	T	0.110	0.061	0.010	2.10E-09	NA
rs34990153	8	9996389	A	G	0.561	0.038	0.005	1.99E-16	MSRA
rs2409742	8	11069960	C	T	0.507	0.036	0.005	7.34E-15	LINC00529
rs12056338	8	12643055	T	G	0.414	0.031	0.005	2.88E-11	LINC03019

rs17294565	8	14124809	C	A	0.381	0.027	0.005	4.74E-09	SGCZ
rs10096633	8	19830921	C	T	0.880	0.070	0.010	8.70E-13	NA
rs1059592	8	22477778	A	G	0.354	0.027	0.005	2.40E-08	CCAR2
rs17818197	8	25872634	G	A	0.212	0.035	0.006	4.99E-10	EBF2
rs2725371	8	30854033	A	G	0.311	0.037	0.005	2.14E-13	PURG
rs1060731	8	41435225	T	C	0.290	0.042	0.007	3.50E-09	NA
rs13262861	8	41508577	C	A	0.824	0.102	0.006	2.84E-59	NKX6-3,ANK1
rs148766658	8	41552046	C	T	0.040	0.110	0.017	7.90E-11	NA
rs2241896	8	41555473	T	C	0.630	0.046	0.007	4.70E-11	NA
rs62515938	8	57483013	T	C	0.261	0.029	0.005	2.97E-08	LINC00968,RPL37P6
rs11786992	8	95685147	A	C	0.640	0.040	0.007	1.70E-09	NA
rs10097617	8	95961626	T	C	0.480	0.037	0.005	1.93E-16	NDUFAF6
rs34340810	8	105661926	G	C	0.927	0.054	0.009	4.08E-10	ZFPM2
rs4734193	8	110140564	C	A	0.532	0.034	0.006	3.70E-08	NUDCD1,TRHR
rs2737226	8	116639474	T	C	0.389	0.038	0.005	2.52E-16	TRPS1
rs11558471	8	118185733	A	G	0.684	0.103	0.005	3.98E-98	SLC30A8
rs17772814	8	128711742	G	A	0.915	0.075	0.013	3.44E-09	CASC11
rs1561927	8	129568078	C	T	0.282	0.035	0.005	8.61E-12	LINC00824
rs3757969	8	145551199	G	C	0.376	0.048	0.005	3.60E-22	DGAT1,SCRT1
rs2294120	8	146003567	A	G	0.544	0.044	0.008	1.62E-08	ZNF34
rs756145	9	1039939	A	G	0.309	0.029	0.005	5.52E-09	H3P29,LINC01230
rs10974438	9	4291928	C	A	0.356	0.047	0.005	8.98E-23	GLIS3
rs10963942	9	19080352	G	A	0.397	0.037	0.005	4.49E-15	HAUS6
rs7867635	9	20241069	C	T	0.411	0.037	0.006	4.21E-09	MLLT3,SLC24A2
rs1063192	9	22003367	A	G	0.560	0.058	0.006	2.90E-19	NA
rs10811660	9	22134068	G	A	0.828	0.239	0.010	1.40E-115	CDKN2A/B
rs7018475	9	22137685	G	T	0.270	0.110	0.007	6.30E-48	NA
rs11793831	9	23362311	T	G	0.405	0.027	0.005	4.21E-09	LINC01239,SUMO2P2
rs1412234	9	28410683	C	T	0.319	0.044	0.005	4.00E-19	LINGO2
rs12001437	9	34074476	C	T	0.373	0.034	0.005	6.27E-13	DCAF12,RN7SKP114
rs1929883	9	81344701	G	A	0.580	0.039	0.006	4.84E-10	PSAT1,MTND2P8
rs17791513	9	81905590	A	G	0.930	0.100	0.013	2.90E-14	NA
rs2796441	9	84308948	G	A	0.591	0.059	0.005	9.96E-37	TLE1-DT
rs555784	9	85318704	T	A	0.616	0.030	0.005	1.70E-10	MTCO3P40,RPS6P12

rs10821311	9	96943059	A	G	0.319	0.036	0.005	4.00E-13	MIRLET7A1HG,LINC02603
rs7046845	9	97804641	A	C	0.910	0.047	0.008	2.26E-08	AOPEP
rs7858727	9	111936128	C	A	0.223	0.034	0.006	8.33E-10	EPB41L4B
rs1431819	9	116943357	G	A	0.689	0.029	0.005	6.33E-09	COL27A1
rs7026688	9	125975397	G	A	0.863	0.044	0.007	3.40E-11	STRBP
rs1752169	9	126586563	A	C	0.270	0.032	0.005	1.64E-09	DENND1A
rs495203	9	136145240	T	C	0.335	0.049	0.005	1.03E-24	ABO
rs448918	9	136885979	A	G	0.287	0.033	0.005	1.11E-09	BRD3OS,VAV2
rs28429551	9	139243334	A	T	0.740	0.073	0.006	9.51E-40	GPSM1
rs11257655	10	12307894	T	C	0.219	0.091	0.007	7.25E-35	CDC123,RN7SL198P
rs878017	10	13566204	A	G	0.541	0.035	0.006	1.13E-08	BEND7
rs36051838	10	34018730	C	T	0.089	0.044	0.008	4.54E-08	LINC02628,LINC00838
rs12263348	10	65305252	T	C	0.339	0.028	0.005	7.77E-09	REEP3
rs10998304	10	70342775	C	T	0.452	0.031	0.005	1.77E-11	TET1
rs177045	10	71321279	G	A	0.316	0.068	0.007	6.60E-18	NEUROG3
rs2642588	10	71466578	G	T	0.702	0.049	0.007	2.20E-14	NEUROG3
rs827237	10	72648336	T	C	0.205	0.037	0.006	3.56E-10	SGPL1
rs2675662	10	75599127	A	G	0.564	0.027	0.005	8.30E-09	CAMK2G
rs7099048	10	77647107	A	G	0.504	0.028	0.005	8.93E-10	LRMDA
rs703981	10	80942855	G	C	0.544	0.061	0.005	2.13E-40	ZMIZ1
rs11201999	10	88124501	C	T	0.537	0.026	0.005	1.25E-08	GRID1
rs10788575	10	89768584	A	G	0.149	0.035	0.006	3.71E-08	PTEN,MED6P1
rs7071943	10	93956552	G	T	0.653	0.044	0.005	2.22E-19	CPEB3
rs7084673	10	94167087	A	G	0.390	0.046	0.007	3.20E-12	NA
rs77014180	10	94307157	G	A	0.060	0.089	0.014	9.60E-11	NA
rs1111875	10	94462882	C	T	0.592	0.092	0.005	5.21E-89	HHEX,Y_RNA
rs146935743	10	94466064	C	T	0.970	0.170	0.030	4.00E-08	HHEX/IDE
rs10882891	10	99059645	C	A	0.415	0.031	0.005	1.68E-11	Metazoa_SRP,RPL12P27
rs2862954	10	101912064	T	C	0.517	0.029	0.005	1.56E-10	ERLIN1
rs2250301	10	104548393	G	A	0.748	0.032	0.005	1.27E-09	WBP1L
rs10787287	10	112647195	T	C	0.759	0.036	0.006	6.56E-11	PDCD4
rs1927157	10	114635381	C	T	0.760	0.064	0.008	2.90E-17	NA
rs114222749	10	114668249	T	C	0.023	0.170	0.021	9.20E-16	NA
rs116425039	10	114681965	G	A	0.990	0.280	0.036	1.00E-14	NA

rs116859590	10	114752410	T	C	0.026	0.240	0.021	3.70E-29	NA
rs34872471	10	114754071	C	T	0.300	0.310	0.007	5.63E-144	NA
rs78025551	10	114757956	C	G	0.850	0.150	0.009	8.40E-63	NA
rs61872774	10	114765390	A	G	0.013	0.280	0.030	2.80E-21	NA
rs10885404	10	114773068	G	T	0.820	0.150	0.018	6.50E-17	TCF7L2
rs141241414	10	114775551	A	G	0.990	0.190	0.028	3.50E-11	NA
rs116369954	10	114793572	C	T	0.030	0.290	0.018	2.10E-56	NA
rs11196201	10	114803307	T	A	0.080	0.180	0.012	9.70E-54	NA
rs4918791	10	114830306	G	A	0.630	0.074	0.007	2.20E-25	NA
rs6585206	10	114859251	A	G	0.190	0.066	0.008	9.40E-16	NA
rs11196229	10	114866172	G	A	0.750	0.092	0.008	6.90E-34	NA
rs10885419	10	114893956	G	C	0.270	0.057	0.007	2.40E-15	NA
rs1225404	10	114914665	T	C	0.640	0.060	0.007	5.00E-19	NA
rs2280141	10	124193181	T	G	0.520	0.045	0.006	1.09E-13	PLEKHA1,ARMS2
rs4929965	11	2197286	A	G	0.385	0.062	0.005	2.46E-38	ASCL2,MIR4686
rs231361	11	2691500	A	G	0.256	0.077	0.007	5.00E-25	KCNQ1
rs2283220	11	2755548	A	G	0.690	0.049	0.007	1.40E-09	KCNQ1
rs2237895	11	2857194	C	A	0.423	0.073	0.005	2.73E-54	KCNQ1
rs2237897	11	2858546	C	T	0.954	0.207	0.017	8.40E-32	KCNQ1
rs10769936	11	8654528	C	T	0.707	0.035	0.005	6.29E-12	TRIM66
rs2403221	11	9852475	A	G	0.657	0.032	0.005	5.60E-11	SBF2
rs117316450	11	14518419	G	C	0.019	0.131	0.018	2.07E-13	COPB1
rs5219	11	17409572	T	C	0.373	0.069	0.005	3.15E-48	KCNJ11
rs62618693	11	32956492	C	T	0.957	0.085	0.011	6.83E-14	QSER1
rs11555762	11	43876698	T	C	0.305	0.041	0.005	8.65E-17	HSD17B12
rs11038672	11	45846498	C	G	0.476	0.029	0.005	1.04E-10	SLC35C1,CRY2
rs7124681	11	47529947	A	C	0.410	0.037	0.006	6.40E-09	NA
rs7929543	11	49351026	C	A	0.083	0.083	0.014	2.20E-09	TYRL
rs116861182	11	55588216	C	A	0.055	0.064	0.011	5.90E-09	OR5D18,OR5L2
rs174541	11	61565908	T	C	0.646	0.029	0.005	8.63E-10	FADS2
rs35169799	11	64031241	T	C	0.066	0.050	0.009	4.79E-08	PLCB3
rs1783541	11	65294799	T	C	0.203	0.048	0.006	3.89E-17	SCYL1
rs144245804	11	69453044	G	A	0.973	0.130	0.015	1.13E-17	CCND1,LINC01488
rs11602873	11	72460762	A	T	0.844	0.098	0.006	8.91E-52	ARAP1

rs480840	11	74625997	C	T	0.423	0.025	0.005	4.46E-08	XRRA1
rs10899283	11	76505202	C	T	0.771	0.031	0.006	1.46E-08	TSKU
rs10830963	11	92708710	G	C	0.278	0.089	0.005	4.57E-68	MTNR1B
rs57235767	11	93013531	C	T	0.710	0.046	0.007	6.60E-11	NA
rs10893829	11	128042575	T	C	0.853	0.058	0.010	1.30E-10	ETS1
rs10750397	11	128234144	A	G	0.290	0.048	0.005	1.01E-20	LINC02098,ETS1
rs11221333	11	128383687	T	C	0.220	0.053	0.008	4.20E-12	NA
rs11063029	12	4301301	T	C	0.057	0.089	0.014	1.70E-10	NA
rs11063069	12	4374373	G	A	0.210	0.056	0.008	7.40E-13	NA
rs3217792	12	4384696	C	T	0.913	0.113	0.011	2.60E-21	CCND2
rs76895963	12	4384844	T	G	0.980	0.482	0.027	1.40E-69	CCND2
rs3217860	12	4399050	G	A	0.258	0.049	0.007	3.90E-09	CCND2
rs12299509	12	4406281	G	A	0.479	0.047	0.007	2.09E-10	CCND2
rs67013744	12	6681786	G	A	0.160	0.035	0.006	4.11E-08	CHD4
rs10841868	12	21781246	G	T	0.739	0.032	0.005	1.65E-09	GYS2,LDHB
rs11048458	12	26465585	T	C	0.250	0.046	0.005	4.66E-18	ITPR2-AS1
rs10771372	12	27962260	C	T	0.805	0.072	0.006	2.20E-35	RN7SKP15,PTHLH
rs10771813	12	31367856	C	A	0.544	0.026	0.005	2.60E-08	DDX11,RPL13AP22
rs10844518	12	33410780	G	A	0.287	0.033	0.005	8.80E-11	ASS1P14,SYT10
rs2733289	12	41838235	C	T	0.478	0.030	0.005	3.98E-11	PDZRN4
rs11181613	12	43046449	C	A	0.855	0.043	0.007	1.37E-10	LINC02451
rs2732480	12	48736303	C	A	0.565	0.034	0.005	3.05E-13	ZNF641
rs7132908	12	50263148	A	G	0.390	0.033	0.005	1.41E-12	FAIM2
rs1872635	12	54541750	A	G	0.686	0.028	0.005	1.28E-08	SMUG1
rs2583921	12	66170481	C	A	0.092	0.095	0.008	8.98E-33	RPSAP52
rs1042725	12	66358347	T	C	0.490	0.054	0.006	1.60E-17	NA
rs1705263	12	71523043	C	A	0.569	0.040	0.005	1.22E-17	TSPAN8
rs11107116	12	93978504	T	G	0.220	0.047	0.009	3.75E-08	SOCS2
rs11108094	12	95928113	A	C	0.068	0.060	0.009	1.16E-10	USP44
rs2197973	12	95928560	T	C	0.538	0.039	0.007	3.60E-08	USP44
rs113036477	12	97848227	C	T	0.938	0.072	0.010	4.09E-13	RMST
rs3764002	12	108618630	C	T	0.741	0.040	0.005	2.69E-14	WSCD2
rs34965774	12	118412373	A	G	0.138	0.052	0.007	1.38E-14	RFC5,KSR2
rs117389214	12	121119057	C	T	0.050	0.088	0.015	6.50E-09	NA

rs73226260	12	121380541	G	A	0.967	0.120	0.019	7.00E-11	NA
rs1800574	12	121416864	T	C	0.030	0.160	0.019	8.90E-17	NA
rs56348580	12	121432117	G	C	0.696	0.058	0.005	2.33E-30	HNF1A
rs12820906	12	123493123	A	G	0.756	0.043	0.006	2.21E-15	PITPNM2
rs12823740	12	124458002	C	A	0.661	0.041	0.005	1.48E-17	ZNF664,RFLNA
rs825476	12	124568456	T	C	0.581	0.052	0.007	6.80E-13	ZNF664-FAM101A
rs11830243	12	132544694	T	C	0.113	0.044	0.007	2.10E-09	EP400
rs11614914	12	133070294	T	C	0.325	0.039	0.005	4.65E-15	FBRSL1
rs12305809	12	133777466	G	A	0.607	0.033	0.005	1.45E-12	ZNF268
rs314879	13	23309382	C	T	0.226	0.039	0.006	5.74E-12	DDX39AP1,SNORD36
rs34584161	13	26776999	A	G	0.761	0.052	0.005	3.54E-22	RNF6
rs9319382	13	28245127	C	T	0.679	0.028	0.005	2.87E-08	POLR1D
rs3742305	13	31036642	C	G	0.729	0.030	0.005	1.51E-08	HMGB1
rs576674	13	33554302	G	A	0.185	0.061	0.006	1.25E-23	KL,TOMM22P3
rs4397977	13	41688401	A	G	0.344	0.029	0.005	3.05E-09	RN7SL597P,MIR3168
rs9316500	13	51094114	T	G	0.708	0.047	0.005	9.97E-21	DLEU7,DLEU1
rs9563574	13	58656599	T	C	0.823	0.041	0.006	1.64E-11	RNA5SP30,LINC02338
rs9563615	13	59077406	A	T	0.710	0.049	0.007	6.40E-11	SRGAP2D
rs11616380	13	80705315	G	T	0.721	0.079	0.005	7.99E-54	LINC01080,SPRY2
rs1475655	13	91963080	A	T	0.747	0.044	0.005	2.47E-16	PPIAP23,MIR17HG
rs9555581	13	109944192	C	T	0.610	0.030	0.005	3.72E-10	LINC00370
rs8005994	14	29744532	A	G	0.655	0.027	0.005	4.52E-08	RNU11-5P,LINC02326
rs17522122	14	33302882	T	G	0.476	0.034	0.005	1.27E-13	AKAP6
rs799661	14	35390146	C	T	0.871	0.043	0.007	7.11E-09	IGBP1P1,BAZ1A-AS1
rs8018512	14	38818723	G	A	0.742	0.037	0.005	1.30E-12	KRT8P1,CLEC14A
rs2933211	14	47313541	A	G	0.501	0.027	0.005	6.24E-09	MDGA2
rs10137475	14	58797953	G	A	0.426	0.026	0.005	4.43E-08	ARID4A
rs4899280	14	69526307	T	C	0.330	0.028	0.005	1.02E-08	DCAF5
rs8008540	14	74948180	C	T	0.569	0.030	0.005	7.99E-11	NPC2
rs2056857	14	77300863	C	T	0.594	0.026	0.005	2.59E-08	LRRC74A
rs10145154	14	79939525	T	C	0.216	0.055	0.006	1.06E-22	NRXN3
rs8010382	14	91963722	G	A	0.437	0.032	0.005	5.74E-12	PPP4R3A
rs73347525	14	101255172	A	G	0.810	0.049	0.008	7.36E-09	MEG3
rs12890750	14	103860309	G	T	0.639	0.028	0.005	2.84E-09	MARK3

rs11073147	15	36392562	G	A	0.540	0.025	0.005	4.26E-08	LINC02853,COX6CP4
rs8032939	15	38834033	C	T	0.250	0.043	0.007	8.40E-09	NA
rs34715063	15	38873115	C	T	0.124	0.095	0.012	2.30E-19	RASGRP1
rs11639470	15	39639171	C	G	0.547	0.027	0.005	1.34E-08	LINC02915,THBS1
rs484943	15	40398754	T	C	0.318	0.033	0.005	7.61E-11	BMF
rs2289739	15	41801512	T	G	0.350	0.050	0.007	1.57E-14	LTK
rs74804697	15	52588722	C	G	0.957	0.084	0.012	4.68E-12	MYO5C,MYO5A
rs75332279	15	53099306	C	T	0.096	0.056	0.008	4.93E-12	RPSAP55,ONECUT1
rs2435907	15	57333416	A	G	0.589	0.029	0.005	1.32E-09	TCF12
rs8033609	15	60938816	A	C	0.545	0.027	0.005	1.11E-08	RORA
rs7163757	15	62391608	C	T	0.574	0.041	0.005	1.64E-18	NPM1P47,C2CD4B
rs34143602	15	63940058	G	A	0.422	0.035	0.005	1.00E-13	HERC1
rs1874832	15	67260238	G	A	0.168	0.038	0.007	3.22E-09	SMASR,SMAD3-DT
rs4776970	15	68080886	A	T	0.638	0.029	0.005	6.14E-10	MAP2K5
rs12917449	15	74331659	C	A	0.197	0.036	0.006	4.71E-10	PML
rs6495182	15	75814388	C	T	0.748	0.041	0.005	2.90E-14	PTPN9
rs12910361	15	77782335	G	A	0.698	0.072	0.005	2.97E-44	LINGO1,HMG20A
rs36111056	15	83461873	G	A	0.780	0.034	0.006	1.97E-09	FSD2
rs8031576	15	90380214	C	A	0.289	0.057	0.005	1.74E-29	ARPIN-AP3S2,AP3S2
rs2290203	15	91512067	A	G	0.202	0.056	0.006	1.79E-22	PRC1,PRC1-AS1
rs55857387	16	300388	T	C	0.805	0.052	0.006	6.71E-19	FAM234A
rs4984980	16	968292	A	G	0.188	0.035	0.006	3.79E-09	LMF1
rs12933120	16	3634746	A	C	0.139	0.042	0.007	3.18E-10	SLX4
rs9927842	16	15153717	T	C	0.162	0.038	0.007	6.84E-09	PDXDC1
rs62034975	16	20392415	C	G	0.300	0.031	0.005	6.60E-10	PDILT
rs7188071	16	28917644	T	C	0.364	0.029	0.005	1.33E-09	RABEP2
rs8054556	16	29958216	A	G	0.462	0.036	0.005	3.68E-15	TMEM219
rs7203521	16	53769293	A	G	0.610	0.041	0.007	3.30E-10	NA
rs1421085	16	53800954	C	T	0.414	0.118	0.005	5.63E-144	FTO
rs62033401	16	53814470	C	T	0.870	0.057	0.010	5.00E-09	NA
rs6499646	16	53843533	T	C	0.920	0.070	0.012	4.80E-09	NA
rs2032912	16	69568303	G	T	0.584	0.042	0.005	2.11E-19	CYB5B,NFAT5
rs72802342	16	75234872	C	A	0.921	0.115	0.009	5.58E-38	ZFP1,CTRB2
rs2925979	16	81534790	T	C	0.308	0.045	0.005	1.54E-19	CMIP

rs11646052	16	85716463	G	A	0.392	0.026	0.005	2.61E-08	GINS2
rs11117364	16	88132199	G	A	0.666	0.029	0.005	7.71E-09	LINC02182,BANP
rs12920022	16	89564055	A	T	0.160	0.039	0.007	2.26E-09	SPG7
rs11870735	17	481604	T	C	0.182	0.034	0.006	1.68E-08	VPS53
rs8071043	17	3988451	C	T	0.328	0.054	0.005	5.16E-28	ZZEF1
rs2243102	17	4839149	C	T	0.422	0.026	0.005	4.94E-08	SLC25A11,GP1BA
rs858519	17	7531965	T	C	0.444	0.026	0.005	1.88E-08	SHBG
rs7219033	17	9787958	A	G	0.314	0.029	0.005	4.94E-09	GLP2R
rs2297508	17	17715317	C	G	0.368	0.033	0.005	1.86E-12	SREBF1
rs7220340	17	27566326	A	G	0.455	0.027	0.005	4.00E-09	TWF1P1,CRYBA1
rs12602834	17	29637308	G	A	0.391	0.029	0.005	7.09E-10	EVI2B,NF1
rs4796224	17	34842521	G	A	0.474	0.025	0.005	2.71E-08	ZNHIT3
rs11657964	17	36100767	A	G	0.404	0.059	0.005	1.23E-36	HNF1B
rs11078916	17	37746307	T	C	0.296	0.037	0.005	4.84E-13	NEUROD2,CDK12
rs684214	17	40696915	T	C	0.273	0.042	0.005	2.57E-16	HSD17B1P1,NAGLU
rs9900074	17	46124326	G	C	0.925	0.055	0.009	3.24E-10	NFE2L1-DT
rs35895680	17	47060322	C	A	0.686	0.056	0.005	1.42E-28	GIP
rs1451506	17	57407019	A	G	0.127	0.042	0.008	2.78E-08	YPEL2,SNRPGP17
rs4325	17	61563200	C	A	0.539	0.035	0.005	6.92E-14	ACE
rs17631783	17	61687600	C	T	0.737	0.049	0.009	3.95E-08	TACO1
rs11655898	17	62201374	C	T	0.070	0.059	0.010	5.85E-10	ERN1
rs2080090	17	65828371	A	T	0.188	0.053	0.006	7.84E-19	BPTF
rs61736066	17	70645032	G	A	0.917	0.051	0.009	2.08E-09	SLC39A11
rs1656794	17	75386909	G	A	0.716	0.031	0.005	3.58E-09	SEPTIN9
rs62075585	17	76762039	G	A	0.474	0.030	0.005	1.25E-10	CYTH1
rs9912236	17	77895311	C	T	0.750	0.031	0.006	1.56E-08	LINC01979,LINC01978
rs7240767	18	7070642	C	T	0.379	0.037	0.006	5.03E-09	LAMA1
rs11662800	18	13271367	A	G	0.418	0.028	0.005	2.42E-09	LDLRAD4
rs303760	18	21083738	T	C	0.348	0.034	0.005	1.48E-12	RMC1
rs346240	18	40063830	G	A	0.223	0.031	0.006	3.62E-08	LINC00907
rs72926932	18	53050646	C	A	0.078	0.075	0.008	7.58E-20	TCF4
rs17684074	18	54675384	G	C	0.739	0.031	0.005	4.12E-09	WDR7
rs1517037	18	56878274	C	T	0.813	0.038	0.006	3.22E-10	SEC11C,GRP
rs663640	18	57846077	T	C	0.218	0.050	0.006	1.15E-19	RNU4-17P,MC4R

rs12454712	18	60845884	T	C	0.617	0.041	0.005	8.30E-18	BCL2
rs2658746	18	74582340	C	T	0.390	0.030	0.005	9.89E-11	ZNF236
rs35004890	19	1224286	T	G	0.221	0.036	0.006	8.57E-10	STK11
rs12977104	19	4949921	A	G	0.200	0.041	0.006	1.46E-12	UHRF1
rs17175860	19	7235146	G	A	0.192	0.045	0.006	2.55E-14	INSR
rs2115107	19	7968168	A	G	0.392	0.038	0.005	8.45E-16	LRRC8E,MAP2K7
rs11666603	19	12496934	C	T	0.744	0.033	0.006	1.71E-09	RPS29P23,ZNF799
rs3111316	19	13038415	A	G	0.588	0.044	0.005	3.43E-21	FARSA
rs10404726	19	18834514	C	T	0.531	0.028	0.005	2.73E-09	CRTC1
rs58542926	19	19379549	T	C	0.076	0.089	0.009	4.67E-25	TM6SF2
rs4805681	19	31835516	C	T	0.610	0.027	0.005	1.32E-08	TSHZ3
rs10406327	19	33890838	C	G	0.525	0.037	0.005	8.75E-16	PEPD
rs429358	19	45411941	T	C	0.855	0.073	0.007	3.98E-28	APOE
rs10407429	19	46157237	G	A	0.579	0.054	0.005	4.79E-31	RN7SL836P,GIPR
rs2238689	19	46178661	C	T	0.418	0.039	0.005	5.40E-09	GIPR
rs11667244	19	47580185	G	A	0.702	0.035	0.005	2.17E-12	ZC3H4
rs6515236	20	22435749	A	C	0.751	0.050	0.009	3.34E-08	LOC105372562 (FOXA2)
rs2268078	20	32596704	A	G	0.655	0.039	0.005	2.14E-15	RALY
rs17265513	20	39832628	C	T	0.198	0.033	0.006	1.13E-08	ZHX3
rs419842	20	42310811	T	A	0.821	0.041	0.006	7.17E-11	MYBL2
rs12625671	20	42994812	C	T	0.114	0.065	0.007	6.74E-19	HNF4A
rs1800961	20	43042364	T	C	0.035	0.166	0.017	2.30E-22	HNF4A
rs6066138	20	45594711	G	A	0.725	0.045	0.005	1.58E-18	EYA2
rs867489	20	48833957	C	T	0.539	0.031	0.005	1.51E-11	PELATON,CEBPB
rs2426439	20	50999627	C	T	0.632	0.037	0.005	2.08E-14	LINC01524
rs2252221	20	51621922	G	A	0.531	0.025	0.005	4.48E-08	TSHZ2
rs911300	20	57387262	G	A	0.542	0.035	0.005	5.19E-14	MIR296,PIEZO1P2
rs1815591	20	61277014	A	T	0.398	0.034	0.005	5.50E-12	SLCO4A1
rs4809369	20	62470785	G	A	0.555	0.034	0.005	4.27E-13	C20orf181,ZBTB46
rs75756987	21	47767295	G	C	0.888	0.044	0.007	1.61E-09	PCNT
rs75401573	22	29805444	C	T	0.924	0.051	0.009	6.47E-09	RFPL4AP6,AP1B1
rs5753043	22	30588041	C	A	0.898	0.061	0.008	3.76E-14	LIF-AS1,HORMAD2
rs117001013	22	32348841	C	T	0.919	0.047	0.008	1.44E-08	YWHAH
rs133015	22	38572526	C	G	0.560	0.029	0.005	5.18E-10	PLA2G6

rs5751061	22	41593873	G	T	0.614	0.026	0.005	3.15E-08	EP300-AS1,L3MBTL2
rs3747207	22	44324855	A	G	0.221	0.047	0.006	9.26E-18	PNPLA3
rs5771069	22	50435480	G	A	0.505	0.033	0.005	1.78E-12	IL17REL
rs112915006	22	50604696	G	A	0.050	0.092	0.015	7.50E-10	NA

chr, chromosome; pos, position; A1, effect allele; A2, other allele; eaf, effect allele frequency

Supplementary Table S4. Quality assessment of each article

1. Are the effect and other alleles coded in the same direction for the exposure and outcome (variable harmonization)?
2. Are the samples used to identify the genetic IVs for the risk factor and outcome drawn from the same ethnic population?
3. Were the two samples independent? (2SMR)
4. Assumption 1-Is there sufficient evidence that the genetic variants are robustly associated with the risk factor of interest?
5. Assumption 2-Are the genetic variants associated with potential confounders? Do the authors present this relationship?
6. Assumption 3-Is there any way for the genetic variants to affect the outcome through alternative pathways (horizontal pleiotropy)?
7. Was the analysis restricted to independent variants (that is, pruned of SNPs in linkage disequilibrium) or did the analysis allow for the correlation between variants?
8. Do the authors manually pick and choose which SNPs go into the instrument to tackle pleiotropy? If so, is the approach and justification clear?
9. Do the authors provide sensitivity analyses such as MR Egger, weighted median, and mode Mendelian randomisation, or use negative control populations?
10. Do the authors provide the data that they used (especially for Mendelian randomisation analyses conducted at the summary level) in a supplement to allow researchers to reproduce their findings?

Supplementary Table S5. Quality assessment results of the 8 articles

	Østergaard SD, et al	Andrews SJ, et al.	Meng L, et al.	Luo J, et al.	Pan Y, et al.	Dybjer E, et al.	Garfield V, et al.	Thomassen JQ, et al.
Q1	✔	✔	✔	✔	✔	✔	✔	✔
Q2	✔	✔	✔	✔	✔	✔	✔	✔
Q3	✔	✔	✔	✔	✔	✔	✔	✔
Q4	✔	✔	✔	✔	✔	✔	✔	✔
Q5	✔	✔	✔	✔	✔	✔	✔	✔
Q6	✔	✔	✔	✔	✔	✔	✔	✔
Q7	✔	✔	✔	✔	✔	✔	✔	✔
Q8	✔	✔	✔	✔	✔	✔	✔	✔
Q9	✔	✔	✔	✔	✔	✔	✔	✔
Q10	✔	✔	✘	✔	✔	✔	✔	✔

Supplementary Table S6. Summary of harmonisation results across exposure and outcome datasets

Outcome Dataset	Initial SNPs from Exposure	Matched SNPs	Removed for Palindromic Ambiguity	Removed for Strand Mismatch	Final SNPs used
IGAP	512	494	62	0	432
EADB	512	503	12	0	491
UKB	512	504	11	0	493

Supplementary Material S2. Method for calculating p-values from reported odds ratios and confidence intervals

For each study:

- The **log odds ratio** was calculated as:

$$\log(OR)$$

- The **standard error (SE)** of the log(OR) was derived from the 95% confidence interval (CI):

$$\text{SE} = \frac{\log(\text{Upper CI}) - \log(\text{Lower CI})}{2 \times 1.96}$$

- The **z-score** was computed as:

$$z = \frac{\log(\text{OR})}{\text{SE}}$$

- Finally, the **two-sided p-value** was estimated from the standard normal distribution:

$$p = 2 \times \left(1 - \Phi(\quad | z | \quad)\right)$$

where Φ\|*Phi*Φ is the cumulative distribution function of the standard normal distribution.

Supplementary Table S7. Result of MR analysis between type 2 diabetes mellitus and Alzheimer's disease (IGAP dataset)

Method	**nSNP**	**OR**	**95% CI**	**p-value**	**MR Egger intercept**
MR Egger	432	0.929	0.772-1.117	0.433	0.437
Weighted median	432	0.977	0.911-1.048	0.519	
Inverse variance weighted	432	0.989	0.901-1.085	0.812	
Simple mode	432	1.114	0.923-1.345	0.262	
Weighted mode	432	0.986	0.910-1.068	0.726	

SNP, single nucleotide polymorphism; OR, odds ratio; CI: confidence interval; IGAP, The International Genomics of Alzheimer's Project

Supplementary Table S8. Result of MR analysis between type 2 diabetes mellitus (T2DM) and Alzheimer's disease (EDAB dataset)

Method	**nSNP**	**OR**	**95% CI**	**p-value**	**MR Egger intercept**
MR Egger	491	1.009	0.963-1.056	0.718	0.418
Weighted median	491	1.012	0.977-1.048	0.506	
Inverse variance weighted	491	0.992	0.969-1.016	0.517	
Simple mode	491	1.075	0.990-1.166	0.084	
Weighted mode	491	1.028	0.987-1.072	0.185	

SNP, single nucleotide polymorphism; OR, odds ratio; CI: confidence interval; EADB, European Alzheimer & Dementia Biobank consortium

Supplementary Table S9. Result of MR analysis between type 2 diabetes mellitus (T2DM) and Alzheimer's disease (UKB dataset)

Method	**nSNP**	**OR**	**95% CI**	**p-value**	**MR Egger intercept**
MR Egger	493	1.0000	0.999-1.000	0.668	0.623
Weighted median	493	1.0002	1.000-1.001	0.304	
Inverse variance weighted	493	1.0000	1.000-1.000	0.993	
Simple mode	493	0.9999	0.999-1.001	0.761	
Weighted mode	493	1.0001	1.000-1.001	0.593	

SNP, single nucleotide polymorphism; OR, odds ratio; CI: confidence interval; UKB, UK Biobank

Supplementary Table S10. PRISMA checklist for systematic review and meta-analysis

Section and Topic	Item #	Checklist item	Location where item is reported
**TITLE**			
Title	1	Identify the report as a systematic review.	Page 1
**ABSTRACT**			
Abstract	2	See the PRISMA 2020 for Abstracts checklist.	Page 1-2
**INTRODUCTION**			
Rationale	3	Describe the rationale for the review in the context of existing knowledge.	Page 1-2
Objectives	4	Provide an explicit statement of the objective(s) or question(s) the review addresses.	Page 2
**METHODS**			
Eligibility criteria	5	Specify the inclusion and exclusion criteria for the review and how studies were grouped for the syntheses.	Page 3
Information sources	6	Specify all databases, registers, websites, organisations, reference lists and other sources searched or consulted to identify studies. Specify the date when each source was last searched or consulted.	Page 2
Search strategy	7	Present the full search strategies for all databases, registers and websites, including any filters and limits used.	Page 2 and Supplementary Table S1
Selection process	8	Specify the methods used to decide whether a study met the inclusion criteria of the review, including how many reviewers screened each record and each report retrieved, whether they worked independently, and if applicable, details of automation tools used in the process.	Page 3
Data collection process	9	Specify the methods used to collect data from reports, including how many reviewers collected data from each report, whether they worked independently, any processes for obtaining or confirming data from study investigators, and if applicable, details of automation tools used in the process.	Page 2-3
Data items	10a	List and define all outcomes for which data were sought. Specify whether all results that were compatible with each outcome domain in each study were sought (e.g. for all measures, time points, analyses), and if not, the methods used to decide which results to collect.	Page 3
	10b	List and define all other variables for which data were sought (e.g. participant and intervention characteristics, funding sources). Describe any assumptions made about any missing or unclear information.	Page 3
Study risk of bias assessment	11	Specify the methods used to assess risk of bias in the included studies, including details of the tool(s) used, how many reviewers assessed each study and whether they worked independently, and if applicable, details of automation tools used in the process.	Page 3
Effect measures	12	Specify for each outcome the effect measure(s) (e.g. risk ratio, mean difference) used in the synthesis or presentation of results.	Page 3
Synthesis methods	13a	Describe the processes used to decide which studies were eligible for each synthesis (e.g. tabulating the study intervention characteristics and comparing against the planned groups for each synthesis (item #5)).	Page 3
	13b	Describe any methods required to prepare the data for presentation or synthesis, such as handling of missing summary statistics, or data conversions.	Page 3

Section and Topic	Item #	Checklist item	Location where item is reported
	13c	Describe any methods used to tabulate or visually display results of individual studies and syntheses.	Page 3
	13d	Describe any methods used to synthesize results and provide a rationale for the choice(s). If meta-analysis was performed, describe the model(s), method(s) to identify the presence and extent of statistical heterogeneity, and software package(s) used.	Page 3
	13e	Describe any methods used to explore possible causes of heterogeneity among study results (e.g. subgroup analysis, meta-regression).	Page 3
	13f	Describe any sensitivity analyses conducted to assess robustness of the synthesized results.	/
Reporting bias assessment	14	Describe any methods used to assess risk of bias due to missing results in a synthesis (arising from reporting biases).	Page 3
Certainty assessment	15	Describe any methods used to assess certainty (or confidence) in the body of evidence for an outcome.	Page 3
**RESULTS**			
Study selection	16a	Describe the results of the search and selection process, from the number of records identified in the search to the number of studies included in the review, ideally using a flow diagram.	Page 7
	16b	Cite studies that might appear to meet the inclusion criteria, but which were excluded, and explain why they were excluded.	Page 7
Study characteristics	17	Cite each included study and present its characteristics.	Page 7
Risk of bias in studies	18	Present assessments of risk of bias for each included study.	Page 7
Results of individual studies	19	For all outcomes, present, for each study: (a) summary statistics for each group (where appropriate) and (b) an effect estimate and its precision (e.g. confidence/credible interval), ideally using structured tables or plots.	Page 7
Results of syntheses	20a	For each synthesis, briefly summarise the characteristics and risk of bias among contributing studies.	Supplementary Table S4 and S5
	20b	Present results of all statistical syntheses conducted. If meta-analysis was done, present for each the summary estimate and its precision (e.g. confidence/credible interval) and measures of statistical heterogeneity. If comparing groups, describe the direction of the effect.	Page 7
	20c	Present results of all investigations of possible causes of heterogeneity among study results.	Page 7
	20d	Present results of all sensitivity analyses conducted to assess the robustness of the synthesized results.	/
Reporting biases	21	Present assessments of risk of bias due to missing results (arising from reporting biases) for each synthesis assessed.	/
Certainty of evidence	22	Present assessments of certainty (or confidence) in the body of evidence for each outcome assessed.	Page 7
**DISCUSSION**			
Discussion	23a	Provide a general interpretation of the results in the context of other evidence.	Page 13

Section and Topic	Item #	Checklist item	Location where item is reported
	23b	Discuss any limitations of the evidence included in the review.	Page 13
	23c	Discuss any limitations of the review processes used.	/
	23d	Discuss implications of the results for practice, policy, and future research.	Page 13
**OTHER INFORMATION**			
Registration and protocol	24a	Provide registration information for the review, including register name and registration number, or state that the review was not registered.	Page 2
	24b	Indicate where the review protocol can be accessed, or state that a protocol was not prepared.	/
	24c	Describe and explain any amendments to information provided at registration or in the protocol.	Page 2
Support	25	Describe sources of financial or non-financial support for the review, and the role of the funders or sponsors in the review.	Page 16
Competing interests	26	Declare any competing interests of review authors.	Page 16
Availability of data, code and other materials	27	Report which of the following are publicly available and where they can be found: template data collection forms; data extracted from included studies; data used for all analyses; analytic code; any other materials used in the review.	Page 16

This work is licensed under CC BY 4.0 (8).

Supplementary Table S11. STROBE-MR - Strengthening the Reporting of Observational Studies in Epidemiology using Mendelian Randomization: A checklist of recommended reporting items

Item No.	Section		Checklist item	Page No.	Relevant text from manuscript
1	**TITLE and ABSTRACT**		Indicate Mendelian randomization (MR) as the study's design in the title and/or the abstract if that is a main purpose of the study	1	Title and Abstract
	**INTRODUCTION**				
2	**Background**		Explain the scientific background and rationale for the reported study. What is the exposure? Is a potential causal relationship between exposure and outcome plausible? Justify why MR is a helpful method to address the study question	4	Introduction
3	**Objectives**		State specific objectives clearly, including pre-specified causal hypotheses (if any). State that MR is a method that, under specific assumptions, intends to estimate causal effects	5	Introduction
	**METHODS**				
4	**Study design and data sources**		Present key elements of the study design early in the article. Consider including a table listing sources of data for all phases of the study. For each data source contributing to the analysis, describe the following:		
		a)	Setting: Describe the study design and the underlying population, if possible. Describe the setting, locations, and relevant dates, including periods of recruitment, exposure, follow-up, and data collection, when available.	7	Methods- Two-sample MR analysis
		b)	Participants: Give the eligibility criteria, and the sources and methods of selection of participants. Report the sample size, and whether any power or sample size calculations were carried out prior to the main analysis	5	Methods- Systematic Review and Meta-MR
		c)	Describe measurement, quality control and selection of genetic variants	8	Methods- Instrumental variables selection
		d)	For each exposure, outcome, and other relevant variables, describe methods of assessment and diagnostic criteria for diseases	S3 Appendix	Methods- Information of instrumental variables consortiums
		e)	Provide details of ethics committee approval and participant informed consent, if relevant	10	Methods- Outcome Genetic Consortia Data

5	**Assumptions**	Explicitly state the three core IV assumptions for the main analysis (relevance, independence and exclusion restriction) as well assumptions for any additional or sensitivity analysis	11<br>24	Methods- Statistical Methods and Sensitivity Analyses<br>Figure 2
6	**Statistical methods: main analysis**	Describe statistical methods and statistics used		
	a)	Describe how quantitative variables were handled in the analyses (i.e., scale, units, model)	11	Methods- Statistical Methods and Sensitivity Analyses
	b)	Describe how genetic variants were handled in the analyses and, if applicable, how their weights were selected	11	Methods- Statistical Methods and Sensitivity Analyses
	c)	Describe the MR estimator (e.g. two-stage least squares, Wald ratio) and related statistics. Detail the included covariates and, in case of two-sample MR, whether the same covariate set was used for adjustment in the two samples	11	Methods- Statistical Methods and Sensitivity Analyses
	d)	Explain how missing data were addressed	8	Methods- Two-sample MR analysis
	e)	If applicable, indicate how multiple testing was addressed	/	
7	**Assessment of assumptions**	Describe any methods or prior knowledge used to assess the assumptions or justify their validity	9	Methods- Instrumental variables selection
8	**Sensitivity analyses and additional analyses**	Describe any sensitivity analyses or additional analyses performed (e.g. comparison of effect estimates from different approaches, independent replication, bias analytic techniques, validation of instruments, simulations)	9	Methods- Instrumental variables selection
9	**Software and pre-registration**			
	a)	Name statistical software and package(s), including version and settings used	12	Methods- Statistical Methods and Sensitivity Analyses
	b)	State whether the study protocol and details were pre-registered (as well as when and where)	/	/
	**RESULTS**			
10	**Descriptive data**			

		a)	Report the numbers of individuals at each stage of included studies and reasons for exclusion. Consider use of a flow diagram	12<br>23	Findings- Systematic Review and Meta-MR<br>Figure 1
		b)	Report summary statistics for phenotypic exposure(s), outcome(s), and other relevant variables (e.g. means, SDs, proportions)	/	Supplementary Table S7-9
		c)	If the data sources include meta-analyses of previous studies, provide the assessments of heterogeneity across these studies	6	Methods- Systematic Review and Meta-MR
		d)	For two-sample MR:<br>i. Provide justification of the similarity of the genetic variant-exposure associations between the exposure and outcome samples<br>ii. Provide information on the number of individuals who overlap between the exposure and outcome studies	/	Supplementary Table S2
11	**Main results**				
		a)	Report the associations between genetic variant and exposure, and between genetic variant and outcome, preferably on an interpretable scale	13	Findings- Two-Sample MR analysis
		b)	Report MR estimates of the relationship between exposure and outcome, and the measures of uncertainty from the MR analysis, on an interpretable scale, such as odds ratio or relative risk per SD difference	13	Findings- Two-Sample MR analysis
		c)	If relevant, consider translating estimates of relative risk into absolute risk for a meaningful time period	/	/
		d)	Consider plots to visualize results (e.g. forest plot, scatterplot of associations between genetic variants and outcome versus between genetic variants and exposure)	26-28	Figure 2-4
12	**Assessment of assumptions**				
		a)	Report the assessment of the validity of the assumptions	8	Methods- Two-Sample MR analysis- Instrumental variables selection
		b)	Report any additional statistics (e.g., assessments of heterogeneity across genetic variants, such as I^2, Q statistic or E-value)	13-14	Findings- Two-Sample MR analysis

13	**Sensitivity analyses and additional analyses**				
		a)	Report any sensitivity analyses to assess the robustness of the main results to violations of the assumptions	13-14	Findings- Two-Sample MR analysis
		b)	Report results from other sensitivity analyses or additional analyses	/	/
		c)	Report any assessment of direction of causal relationship (e.g., bidirectional MR)	/	/
		d)	When relevant, report and compare with estimates from non-MR analyses	/	/
		e)	Consider additional plots to visualize results (e.g., leave-one-out analyses)	/	/
	**DISCUSSION**				
14	**Key results**		Summarize key results with reference to study objectives	14	Discussion
15	**Limitations**		Discuss limitations of the study, taking into account the validity of the IV assumptions, other sources of potential bias, and imprecision. Discuss both direction and magnitude of any potential bias and any efforts to address them	17	Discussion- Strengths and limitations
16	**Interpretation**				
		a)	Meaning: Give a cautious overall interpretation of results in the context of their limitations and in comparison with other studies	14	Discussion
		b)	Mechanism: Discuss underlying biological mechanisms that could drive a potential causal relationship between the investigated exposure and the outcome, and whether the gene-environment equivalence assumption is reasonable. Use causal language carefully, clarifying that IV estimates may provide causal effects only under certain assumptions	14	Discussion
		c)	Clinical relevance: Discuss whether the results have clinical or public policy relevance, and to what extent they inform effect sizes of possible interventions	16	Discussion-Clinical relevance
17	**Generalizability**		Discuss the generalizability of the study results (a) to other populations, (b) across other exposure periods/timings, and (c) across other levels of exposure	14	Discussion
	**OTHER INFORMATION**				

18	**Funding**	Describe sources of funding and the role of funders in the present study and, if applicable, sources of funding for the databases and original study or studies on which the present study is based	18	Acknowledgements
19	**Data and data sharing**	Provide the data used to perform all analyses or report where and how the data can be accessed, and reference these sources in the article. Provide the statistical code needed to reproduce the results in the article, or report whether the code is publicly accessible and if so, where	12	Methods- Statistical Methods and Sensitivity Analyses
20	**Conflicts of Interest**	All authors should declare all potential conflicts of interest	18	Acknowledgements

This checklist is copyrighted by the Equator Network under the Creative Commons Attribution 3.0 Unported (CC BY 3.0) license (9).

1. Scott RA, Scott LJ, Mägi R, Marullo L, Gaulton KJ, Kaakinen M, et al. An Expanded Genome-Wide Association Study of Type 2 Diabetes in Europeans. Diabetes. 2017;66(11):2888-902.
2. Mahajan A, Wessel J, Willems SM, Zhao W, Robertson NR, Chu AY, et al. Refining the accuracy of validated target identification through coding variant fine-mapping in type 2 diabetes. Nat Genet. 2018;50(4):559-71.
3. Fuchsberger C, Flannick J, Teslovich TM, Mahajan A, Agarwala V, Gaulton KJ, et al. The genetic architecture of type 2 diabetes. Nature. 2016;536(7614):41-7.
4. Sudlow C, Gallacher J, Allen N, Beral V, Burton P, Danesh J, et al. UK biobank: an open access resource for identifying the causes of a wide range of complex diseases of middle and old age. PLoS Med. 2015;12(3):e1001779.
5. Cook JP, Morris AP. Multi-ethnic genome-wide association study identifies novel locus for type 2 diabetes susceptibility. Eur J Hum Genet. 2016;24(8):1175-80.
6. Estrada K, Aukrust I, Bjørkhaug L, Burtt NP, Mercader JM, García-Ortiz H, et al. Association of a low-frequency variant in HNF1A with type 2 diabetes in a Latino population. Jama. 2014;311(22):2305-14.
7. Vujkovic M, Keaton JM, Lynch JA, Miller DR, Zhou J, Tcheandjieu C, et al. Discovery of 318 new risk loci for type 2 diabetes and related vascular outcomes among 1.4 million participants in a multi-ancestry meta-analysis. Nat Genet. 2020;52(7):680-91.
8. Page MJ, McKenzie JE, Bossuyt PM, Boutron I, Hoffmann TC, Mulrow CD, et al. The PRISMA 2020 statement: an updated guideline for reporting systematic reviews. Bmj. 2021;372:n71.
9. Skrivankova VW, Richmond RC, Woolf BAR, Davies NM, Swanson SA, VanderWeele TJ, et al. Strengthening the reporting of observational studies in epidemiology using mendelian randomisation (STROBE-MR): explanation and elaboration. Bmj. 2021;375:n2233.
